# Effects of Low-Carbohydrate and Ketogenic Diets on Aerobic Performance in Trained Athletes: A Systematic Review and Meta-Analysis

**DOI:** 10.3390/nu18050740

**Published:** 2026-02-25

**Authors:** Mateusz Gawelczyk, Magdalena Kaszuba, Adam Zając, Adam Maszczyk

**Affiliations:** Institute of Sport Sciences, Academy of Physical Education, 40-065 Katowice, Poland; m.gawelczyk@awf.katowice.pl (M.G.); m.kaszuba@awf.katowice.pl (M.K.); a.maszczyk@awf.katowice.pl (A.M.)

**Keywords:** fat oxidation, metabolic adaptation, trained athletes, exercise economy, substrate utilization, time to exhaustion, temporal adaptation threshold

## Abstract

Background/Objectives: While traditional sports nutrition emphasizes high carbohydrate intake for endurance athletes, trained athletes may achieve metabolic adaptation to low-carbohydrate and ketogenic diets with maintained or improved performance outcomes. This systematic review and meta-analysis synthesize evidence on the effects of low-carbohydrate (≤130 g·day^−1^ or ≤25% total energy) and ketogenic (<50 g·day^−1^ or <10% total energy) diets on aerobic performance variables in trained athletes. Methods: A comprehensive search of five electronic databases (PubMed, SCOPUS, Web of Science, SPORTDiscus, and Cochrane Central Register of Controlled Trials) identified 33 aerobic-focused studies meeting comprehensive inclusion criteria. Selected studies examined trained athletes (≥6 months structured training, age 18–45 years) randomized to low-carbohydrate, ketogenic, or high-carbohydrate control conditions with outcome data on aerobic performance variables (VO_2_max, time trial performance, time to exhaustion, and exercise economy) and metabolic markers (fat oxidation and substrate utilization). Quality assessment employed Newcastle-Ottawa Scale methodology. Results: Maximal aerobic capacity (VO_2_max) was preserved in 50.0% of studies, with 11.1% documenting improvements. Submaximal exercise economy showed the greatest sensitivity, with 50.0% documenting impaired efficiency. Time to exhaustion demonstrated context-dependent effects, with 69.2% maintaining performance. All 30 studies measuring fat oxidation demonstrated consistent increases (+28% to +200%). Critically, temporal analysis identified a 1-week adaptation threshold: studies measuring outcomes within ≤7 days documented performance impairment, while studies measuring at >1 week consistently demonstrated maintained or improved performance. Conclusions: Low-carbohydrate diets reliably induce metabolic adaptation characterized by dramatically increased fat oxidative capacity. However, aerobic performance responses are nuanced, with preserved maximal aerobic power, transient submaximal efficiency impairments, and context-dependent endurance effects. Adaptation involves initial acute-phase decrements (≤7 days) followed by recovery. Evidence supports periodized carbohydrate strategies balancing metabolic adaptation benefits from low-carbohydrate training phases with carbohydrate requirements during competition.

## 1. Introduction

Dietary macronutrient composition has long been recognized as a critical determinant of athletic performance capacity in trained individuals [1,2]. Traditional sports nutrition paradigms have emphasized high carbohydrate intake (typically 6–10 g·kg^−1^·day^−1^) as a fundamental requirement for optimizing performance in endurance and high-intensity exercise, based on extensive evidence demonstrating that endogenous muscle and hepatic glycogen stores are crucial substrates for ATP resynthesis during moderate-to-high intensity exercise exceeding 70% VO_2_max [3,4,5]. This carbohydrate-centric nutritional framework has been formalized in contemporary sports nutrition guidelines by major professional organizations, establishing high carbohydrate availability as the standard recommendation for competitive athletes [1,5].

However, emerging scientific evidence and practical adoption among elite athletes have challenged this conventional paradigm, demonstrating that trained athletes may adapt to sustained low-carbohydrate dietary interventions with maintained or improved performance outcomes in specific contexts [6,7,8,9]. The physiological capacity of trained skeletal muscle to shift substrate oxidation patterns in response to dietary macronutrient availability, a phenomenon termed “metabolic flexibility”, has become an increasingly recognized adaptation mechanism in sports physiology [3,10]. This capacity reflects the fundamental plasticity of mitochondrial enzymatic systems, which can be substantially remodeled through sustained dietary interventions or training manipulations to enhance fat oxidative capacity and reduce carbohydrate dependence [3,11,12].

Low-carbohydrate dietary interventions are defined as dietary patterns that restrict carbohydrate intake to ≤25% of total energy intake or ≤130 g per day. Ketogenic diets are characterized by severe carbohydrate restriction (less than 50 g per day or less than 10% of total energy intake) and are designed to induce physiological ketosis. These diets operate through distinct metabolic mechanisms that fundamentally reorganize cellular energy substrate utilization [13,14,15]. These interventions bypass the traditional glucose-glycogen-pyruvate-lactate cycle that dominates carbohydrate-replenishment conditions, instead engaging fat oxidative pathways to a substantially greater degree [7,8]. The mechanistic basis for considering low-carbohydrate and ketogenic diets in athletic populations rests on several theoretical presumptions: enhanced fat oxidation capacity through mitochondrial enzyme upregulation including carnitine palmitoyl transferase-I (CPT-I), hydroxyacyl-CoA dehydrogenase (HADH), and acyl-CoA oxidase-1 (ACOX-1), enabling sustained ATP generation from lipid substrates [3,10,12]; reduced reliance on limited glycogen stores, potentially benefiting very long-duration endurance efforts (>4 h) where glycogen depletion otherwise becomes performance-limiting [7,8,16]; diminished exercise-induced metabolic perturbation and inflammation through reduced carbohydrate-induced insulin responses and postprandial hyperglycemia [17,18,19]; as well as preserved or enhanced body composition through reduced fat mass and maintained or improved lean tissue mass [6,13,14].

The transition from carbohydrate-dependent to fat-dependent energy provision requires substantial metabolic reorganization encompassing mitochondrial remodeling, enzymatic adaptations, and alterations in muscle fiber-type-specific substrate preferences. The temporal characteristics of this adaptive process have been the subject of considerable scientific debate. Early mechanistic investigations demonstrated that substantial increases in fat oxidative enzyme activity (CPT-I, HADH, and ACOX-1) occur within 7–14 days of dietary carbohydrate restriction, with maximum adaptive responses manifesting by 4–6 weeks of sustained intervention [3,11,12]. Mitochondrial structural changes, including increased mitochondrial density and cristae elaboration, have similarly been documented across this 4–6-week timeframe [3,10]. However, the time course of performance consequences of these metabolic adaptations, and critically, whether initial transient performance impairment during early adaptation phases is followed by recovery or sustained deficit, has remained incompletely characterized in the performance physiology literature.

The published literature examining low-carbohydrate and ketogenic diet effects on trained athletes’ performance demonstrates striking heterogeneity in reported findings, creating substantial uncertainty for practitioners attempting to evaluate intervention efficacy. Multiple investigations documenting high-quality randomized controlled trials or crossover designs have reported substantial performance impairments with low-carbohydrate diets, including reduced exercise economy (increased VO_2_ cost of submaximal exercise) [20,21,22], impaired time trial performance [23,24], and compromised high-intensity interval training capacity [20,23,25]. Notably, elite athlete populations, including competitive race walkers, have shown pronounced performance deficits of 8% reduced interval training pace [23] and 2.3% slower completion time of 10,000 m race walking [20] when consuming low-carbohydrate, high-fat diets. Furthermore, investigations employing shorter adaptation periods have documented performance impairments that were not subsequently reversed [20,21,22].

Conversely, other rigorous investigations employing comparable or longer intervention periods have documented performance maintenance or improvement with low-carbohydrate and ketogenic diets [6,7,8,9,26,27], particularly in ultra-endurance contexts where very long exercise duration (9–36 months of habitual ketogenic diet consumption) [7,8], metabolic flexibility optimization [26,27], and perhaps superior fat oxidation capacity enable sustained performance or performance gains [6,7]. Several mechanistic investigations have confirmed robust increases in fat oxidation rates (ranging from 34% to 2.3-fold higher increases) following low-carbohydrate dietary interventions [3,4,7,10,16,28,29], establishing that the fundamental metabolic substrate shift occurs consistently and predictably across populations [3,11,12].

This apparent contradiction between comprehensive evidence of metabolic adaptation (universally documented fat oxidation increases) and highly variable performance responses (ranging from impairment to improvement) suggests that performance outcomes depend substantially on intervention characteristics, adaptation duration, individual metabolic responsiveness, or measurement timing relative to adaptive processes. The observation that some investigations measuring performance at 3–4 weeks document impairment [20,21], while others evaluating at 6–12 weeks show maintenance or improvement [6,27], suggests that a critical temporal threshold may separate acute perturbation from chronic adaptation and recovery [8,20,21]. Despite these clinical and research questions, no comprehensive review and meta-analysis synthesizing findings across diverse interventions, populations, and evaluation time points has been conducted to systematically characterize the magnitude, temporal characteristics, and predictors of low-carbohydrate diet effects on the performance of trained individuals.

Emerging evidence suggests substantial interindividual variability in response to low-carbohydrate and ketogenic dietary interventions, with particular populations showing marked response heterogeneity [6,22,25,26,30]. Several investigations have documented divergent performance responses ranging from substantial improvement to severe impairment within single-study cohorts [6,7,22], suggesting that population-level summary statistics may obscure clinically meaningful individual differences. Proposed mechanisms underlying individual variability include genetic variation affecting mitochondrial function and fat oxidative enzyme expression [7,22], baseline metabolic health status, including glucose homeostasis and insulin sensitivity [26,31], autonomic nervous system characteristics reflecting cardiorespiratory and metabolic stress responses [30], and individual tolerance to metabolic perturbation including gastrointestinal symptoms and subjective well-being [6,22,30]. The potential identification of predictive biomarkers or phenotypes that stratify athletes into responder categories (optimal, adequate, poor responders) represents a promising avenue for precision metabolic medicine approaches to dietary intervention personalization [22,26,30]. However, there is limited evidence from research studies on how individuals respond to low-carbohydrate diets. Only five such studies have been published to date. Inconsistent methodology and outcome measurement prevent the quantitative synthesis required to draw firm conclusions regarding response prediction or responder phenotyping.

The low-carbohydrate and ketogenic diet literature is subject to potential publication bias concerns, as researchers with positive findings regarding dietary intervention efficacy may have greater incentive to publish favorable results, while null or negative findings may be suppressed from publication [32,33]. Additionally, funding sources, including diet-related industries, may introduce bias toward favorable outcomes [9,34]. However, previous systematic reviews of low-carbohydrate interventions in athletic populations have not formally evaluated publication bias risk or conducted sensitivity analyses examining effect stability across varying methodological quality tiers or study designs. The high-profile nature of low-carbohydrate diet adoption among elite athletes, combined with intense public and media interest, may paradoxically reduce publication bias by increasing visibility of negative findings, including those documenting performance impairments [20,23]; however, this remains empirically unexamined in the systematic review context.

A critical and largely unresolved question in the low-carbohydrate diet literature concerns the time course of performance adaptation. Do studies measuring performance at different time points relative to dietary intervention initiation (ranging from 2 days to 36 months) capture distinct phases of metabolic and performance adaptation with systematically different outcomes? Several individual investigations suggest performance impairment during acute adaptation phases (≤7 days) followed by recovery of 3–4 weeks [20,21,35], but comprehensive temporal trajectory analysis across multiple investigations remains absent from the literature. The potential identification of critical adaptation thresholds (e.g., one-week time point) separating acute perturbation from chronic adaptation would have substantial implications for research design, athlete counseling, and evidence interpretation.

Given the substantial heterogeneity in published findings, limited evidence regarding individual variability and response prediction, unexamined publication bias concerns, and inadequately characterized temporal adaptation trajectories, a comprehensive synthesis of the available literature is warranted. Accordingly, this systematic review and meta-analysis integrate evidence across diverse low-carbohydrate and ketogenic diet interventions, populations, measurement approaches, and adaptation phases. This approach aims to establish evidence-based conclusions regarding performance effects, identify critical moderating variables, characterize response heterogeneity, and guide future research prioritization.

The primary objectives of this systematic review and meta-analysis were to: (1) quantify performance effects of low-carbohydrate (≤130 g·day^−1^ or ≤25% energy) and ketogenic (<50 g·day^−1^ or <10% energy) diets on aerobic performance variables (maximal oxygen uptake, exercise economy, time to exhaustion, time trial performance) in trained athletes (≥6 months structured training experience, age 18–45 years); (2) document metabolic substrate utilization shifts (fat oxidation rates, respiratory exchange ratios, and carbohydrate oxidation) accompanying low-carbohydrate dietary adaptation to characterize physiological mechanisms underlying performance responses; (3) identify critical temporal adaptation thresholds by stratifying findings according to intervention duration (acute ≤7 days vs. early chronic 8–31 days vs. mid-chronic 4–12 weeks vs. extended chronic >12 weeks) to determine whether distinct adaptation phases produce systematically different performance outcomes; (4) characterize individual variability in performance responses by synthesizing evidence from studies explicitly examining interindividual heterogeneity, identifying predictive biomarkers (heart rate variability, glucose homeostasis, genetic variants) or responder phenotypes; (5) conduct comprehensive quality assessment and bias evaluation including Newcastle-Ottawa Scale quality ratings, publication bias testing (Egger’s regression, Begg’s test, trim-and-fill analysis), and sensitivity analyses examining findings stability across quality tiers, study designs, and sample sizes; and (6) to formulate evidence-based recommendations for athletes, coaches, and practitioners regarding low-carbohydrate diet implementation timing, expected performance trajectories, individual response considerations, and research priorities. This systematic review and meta-analysis were conducted following the Preferred Reporting Items for Systematic Reviews and Meta-Analyses (PRISMA 2020) guidelines to ensure transparency, rigor, and reproducibility of evidence synthesis and to provide comprehensive characterization of low-carbohydrate and ketogenic diet effects on trained athlete performance.

## 2. Methods

### 2.1. Study Design and Search Strategy

This systematic review and meta-analysis were conducted following the Preferred Reporting Items for Systematic Reviews and Meta-Analyses (PRISMA 2020) guidelines [36]. The review protocol was prospectively registered in the PROSPERO database (CRD420261277181) under the title “Effects of Low-Carbohydrate and Ketogenic Diets on Aerobic Performance in Trained Athletes: A Systematic Review and Meta-Analysis”. A comprehensive semantic search was conducted across five major electronic databases: PubMed/MEDLINE, Web of Science, Scopus, SPORTDiscus, and the Cochrane Central Register of Controlled Trials. Additionally, preprint repositories (bioRxiv, medRxiv) and gray literature sources were screened for relevant unpublished data. However, no preprints meeting our inclusion criteria were identified, and all 33 studies included in the final analysis were peer-reviewed publications. Additionally, preprint repositories (bioRxiv, medRxiv) and gray literature sources were screened for relevant unpublished data. Our team was employed to retrieve the 500 papers most relevant to the research question on low-carbohydrate and ketogenic diets and exercise performance from across 850 academic papers indexed in the database, after which studies were filtered to retain only those reporting aerobic performance and metabolic outcomes in trained athletes.

The semantic search query employed standardized Boolean operators and Medical Subject Headings (MeSH) and free-text keywords in various combinations:

(“ketogenic diet” OR “low-carbohydrate diet” OR “LCHF” OR “keto diet” OR “low-CHO” OR “low-carb”) AND (“athletic performance” OR “exercise performance” OR “VO_2_max” OR “maximal oxygen uptake” OR “lactate threshold” OR “time to exhaustion” OR “running economy” OR “cycling economy”) AND (“athletes” OR “trained” OR “endurance” OR “competitive” OR “elite”) AND (“randomized” OR “RCT” OR “crossover” OR “trial” OR “intervention”).

Boolean operators (AND, OR) were employed to refine search specificity. Database filters restricted results to peer-reviewed studies published in the English language. Manual screening of reference lists from included studies and relevant reviews was performed to identify additional eligible investigations. The search strategy was conducted through October 2025.

### 2.2. Eligibility Criteria and Study Selection

#### 2.2.1. Population

Studies were included if they enrolled trained athletes, defined as individuals possessing ≥6 months of regular structured training experience in their respective sport or discipline. The age range was restricted to 18–45 years to ensure applicability to adult populations. No restrictions were placed on participant sex, and studies including male-only, female-only, or mixed-sex cohorts were eligible for inclusion. Minimum baseline training characteristics included documented competitive participation, training histories, or VO_2_max values indicating trained status (VO_2_max > 40 mL·kg^−1^·min^−1^ for endurance athletes). Studies enrolling untrained individuals, recreational exercisers, or sedentary populations were excluded. Studies including mixed populations (trained and untrained) were excluded if separate data for trained subgroups could not be extracted.

#### 2.2.2. Intervention (I)

Studies were included if they examined low-carbohydrate dietary interventions defined as ≤130 g·day^−1^ total carbohydrate intake or ≤25% energy from carbohydrates, or ketogenic diet interventions defined as <50 g carbohydrates·day^−1^ or <10% energy from carbohydrates with confirmed physiological ketosis (β-hydroxybutyrate ≥ 0.5 mmol·L^−1^). Intervention duration required ≥3 days for acute dietary manipulations or ≥4 weeks for chronic adaptation protocols. Studies documenting dietary compliance through objective measures (food diaries, urinary ketones, serum ketone bodies, and macronutrient analysis) or well-documented adherence protocols were included. Studies involving supplementation combined with dietary intervention, training modifications concurrent with diet manipulation, or other confounding co-interventions were excluded if dietary effects could not be isolated.

#### 2.2.3. Comparison

Studies were included if they incorporated appropriate control or comparison conditions, including high-carbohydrate diets (>60% energy from carbohydrates or >300 g·day^−1^), balanced/mixed diets (45–55% energy from carbohydrates), or habitual dietary intake. Studies employing within-subject comparisons (crossover designs) comparing identical individuals under low-carbohydrate and control conditions were included. Studies without explicit control groups were excluded.

#### 2.2.4. Outcomes

Studies were included if they measured a minimum of two variables encompassing the following domains. Aerobic performance variables included maximal oxygen uptake (VO_2_max, VO_2_peak) measured in mL·kg^−1^·min^−1^ or L·min^−1^, lactate threshold power expressed in watts (W), heart rate response (beats·min^−1^), or VO_2_ at threshold, time to exhaustion (TTE) in minutes at standardized exercise intensity, heart rate measures including resting, submaximal at defined intensity, and maximal responses, running economy or cycling economy quantified as VO_2_ cost per unit distance, respiratory exchange ratio (RER) or respiratory quotient (RQ), and time trial performance including 5 km running distances, cycling time trials, and race walking completion times or distances. Metabolic variables included blood lactate concentration (mmol·L^−1^) measured at rest, during submaximal exercise, or at peak exercise intensity, base excess (mmol·L^−1^); fat oxidation rates quantified in g·min^−1^ or as a percentage of total energy oxidation; carbohydrate oxidation rates in g·min^−1^ or as a percentage of total energy oxidation; and muscle glycogen concentration expressed in mmol·kg^−1^ dry muscle mass.

#### 2.2.5. Study Design

Eligible study designs included randomized controlled trials (RCT), randomized crossover trials, parallel group designs, and repeated-measures crossover protocols. Minimum methodological requirements included: (1) prospective study design; (2) randomized allocation to intervention sequence (for crossover designs); (3) appropriate washout periods (≥48 h) between intervention conditions in crossover designs; (4) quantifiable outcome data reported as means and standard deviations or means with 95% confidence intervals; (5) peer-review publication status; and (6) sufficient methodological detail for quality assessment. While preprint repositories were searched to ensure comprehensive coverage of the literature, ultimately, no preprints were included in the analysis. All 33 of the included studies were published in peer-reviewed journals.

Studies were excluded if they: (1) enrolled untrained or recreationally active individuals; (2) lacked control or comparison group; (3) provided no confirmation of dietary adherence; (4) included animal studies, case reports, or conference abstracts without subsequent peer-reviewed publication; (5) combined dietary intervention with confounding nutritional supplements, co-prescribed medications, or concurrent training modifications that prevented isolation of dietary effects; (6) lacked complete numerical data (means ± SD or 95% CI) necessary for meta-analysis; or (7) were published in languages other than English.

### 2.3. Study Selection Process

The initial semantic database search across five electronic databases and preprint repositories identified 500 records. After removal of duplicates and application of automated filtering tools based on language and publication type, 190 unique records remained for title and abstract screening. Title and abstract screening were performed by two independent reviewers (Kappa agreement = 0.78) using standardized screening criteria covering population, intervention, study design, comparator/control condition, aerobic performance and metabolic outcomes, study duration, publication quality, dietary compliance, and absence of confounding co-interventions. Reviewers applied a holistic screening approach, integrating all criteria simultaneously rather than using sequential threshold-based filtering. Forty-nine records were identified as potentially meeting eligibility criteria and were retrieved for full-text assessment.

Of the 49 full-text articles reviewed, 33 studies that met the comprehensive inclusion criteria for aerobic performance and metabolic outcomes in trained athletes were included in the final systematic review. Sixteen studies were excluded from the primary analysis because their primary outcomes were anaerobic or mixed (e.g., power-related outcomes, one-repetition maximum strength, Wingate tests, vertical jump/countermovement jump, or repeated sprint ability) or because it was not possible to isolate aerobic outcomes from anaerobic performance measures. Meta-analyses examining specific aerobic performance and metabolic variables were based on all 33 aerobic-focused studies with complete data extraction for both aerobic performance and metabolic variables. All included studies were peer-reviewed journal publications. Although preprint repositories (bioRxiv, medRxiv) were systematically searched, no preprints meeting our eligibility criteria, specifically the requirement for peer-review publication status, were identified for inclusion.

### 2.4. Data Extraction and Quality Assessment

Two independent reviewers conducted data extraction using standardized data collection forms developed a priori. Extracted variables included the following: (1) study identification—author names, publication year, and study design classification; (2) population characteristics—sample size (total and per group), participant demographics (age range, mean ± SD), training status/sport discipline, and baseline fitness measures; (3) intervention characteristics—dietary type (low-carbohydrate vs. ketogenic), carbohydrate restriction magnitude (g·day^−1^ or % energy), fat percentage, protein percentage, total energy intake, duration of intervention, and dietary compliance verification methods; (4) comparison/control diet composition and duration; (5) study methodology—design (crossover, parallel, RCT), washout period duration (if applicable), randomization methods, and blinding status; (6) aerobic performance and metabolic outcome measures—specific variables measured (VO_2_max, time to exhaustion, time trial performance, exercise economy, respiratory exchange ratio, fat oxidation, and blood lactate), exercise protocols (intensity, duration, modality, and equipment), and timing of measurements relative to dietary intervention; (7) key results—baseline and post-intervention values for all measured variables, effect sizes or percentage changes, statistical significance (*p*-values, 95% confidence intervals), and direction of effect (improvement, impairment, and no change); (8) adaptation timeline—duration of dietary intervention prior to performance testing, evidence of metabolic adaptation (ketosis levels, substrate utilization changes), and acute versus chronic effect documentation; and (9) study quality and limitations—quality scores, potential confounding factors, generalizability issues, and methodological concerns.

Discrepancies in data extraction between reviewers were resolved through consensus discussion with a third reviewer, representing systematic verification of aerobic-specific outcomes. A large language model was employed to systematically extract structured data from published studies according to detailed extraction instructions for each data column, followed by human verification of automated extractions for accuracy.

The Newcastle–Ottawa Scale (NOS) was employed to evaluate study quality and bias risk. The NOS assesses three domains: (1) selection bias—adequacy of case definition, representativeness of participants, selection of controls, and control definition (maximum 4 points); (2) comparability of groups—matching on important factors and assessment of additional confounders (maximum 2 points); and (3) outcome assessment—blinding of outcome assessors, adequacy of follow-up, and completeness of outcome data (maximum 3 points). Total NOS scores ranged from 0 to 9, with scores ≥7 classified as high quality, scores 5–6 classified as moderate quality, and scores <5 classified as low quality. The mean NOS score for the 33 aerobic-focused included studies was 7.2 ± 1.1, indicating overall high methodological quality.

Additional quality considerations included assessment of: (1) randomization adequacy and concealment; (2) blinding of participants, researchers, and outcome assessors; (3) completeness of outcome data and dropout reporting; (4) selective outcome reporting; (5) sample size adequacy and power calculations; (6) baseline characteristic balance between groups; (7) adequate intervention adherence monitoring; (8) appropriateness of statistical analyses; and (9) potential conflicts of interest.

### 2.5. Statistical Analysis

Statistical analyses were conducted using Review Manager (RevMan, version 5.4.1, The Cochrane Collaboration, London, UK), Stata (version 16.0, StataCorp LLC, College Station, TX, USA), and the ‘meta’ and ‘metafor’ packages in R (version 4.2.2).

#### 2.5.1. Effect Size Computation

For binary outcomes (improved vs. maintained vs. impaired performance), results were coded as categorical variables and tabulated. For continuous outcomes, pooled effect sizes were calculated as standardized mean differences (Cohen’s d) or odds ratios (OR) with 95% confidence intervals (CI) depending on outcome type. When studies reported multiple measures of the same construct (e.g., time trial performance at different distances), the primary outcome was selected a priori to avoid unit-of-analysis errors. When studies reported results for multiple performance domains (aerobic, anaerobic, metabolic), outcomes were analyzed within corresponding domains to prevent ecological correlation artifacts.

#### 2.5.2. Heterogeneity Assessment

Statistical heterogeneity was assessed using Cochran’s Q test (significance at *p* < 0.05) and the I^2^ statistic (percentage of variability attributable to heterogeneity rather than chance). I^2^ values were interpreted as follows: I^2^ < 25% indicating low heterogeneity, 25–75% indicating moderate heterogeneity, and >75% indicating high heterogeneity. When low heterogeneity was observed (I^2^ < 25%, *p* > 0.05), fixed-effects models were employed for meta-analysis. When moderate to high heterogeneity was observed (I^2^ ≥ 25%, *p* < 0.05), random-effects models (DerSimonian and Laird) were employed to account for between-study variance.

#### 2.5.3. Primary Meta-Analysis

Comprehensive meta-analyses were conducted examining effects of low-carbohydrate/ketogenic diets on 33 aerobic-focused studies, organized across three primary outcome domains: aerobic performance outcomes encompassing maximal oxygen uptake (VO_2_max; 18 studies), submaximal exercise economy (4 studies), and time to exhaustion and endurance capacity (13 studies); metabolic variables including fat oxidation rates (30 studies) and substrate utilization patterns (14 studies); and temporal adaptation analysis examining acute versus chronic adaptation effects (6 studies) and intervention duration effects (8 studies). Subgroup analyses were stratified by intervention type (ketogenic < 50 g CHO/day versus low-carbohydrate ≤ 130 g CHO/day versus high-fat without strict carbohydrate restriction), intervention duration (acute ≤ 7 days versus chronic 8–31 days versus extended >31 days), athlete population (endurance athletes versus mixed trained athletes), and study design (crossover versus parallel group versus randomized controlled trials).

#### 2.5.4. Publication Bias Assessment

Publication bias was evaluated using the following: (1) visual inspection of funnel plots (effect size vs. standard error); (2) Egger’s regression test (asymmetry test, *p* < 0.05 indicating potential asymmetry); (3) Begg’s test (rank correlation test); and (4) trim-and-fill analysis (imputing theoretically missing studies to assess impact on pooled estimates). Small sample correction methods were applied when studies with *n* < 15 per group were included.

#### 2.5.5. Sensitivity Analyses

Sensitivity analyses assessed robustness of findings by: (1) sequentially excluding each study and recalculating pooled effect sizes to identify disproportionate influencers; (2) excluding studies with low NOS quality scores (<7) to examine high-quality evidence subgroup; (3) excluding studies employing exclusively crossover designs to assess potential carryover effects; (4) excluding studies with intervention durations <3 days to examine acute vs. chronic distinctions; and (5) restricting analyses to studies with ≥20 participants per group to exclude small sample studies.

#### 2.5.6. Adaptation Timeline Analysis

A temporal trajectory analysis was conducted examining acute (≤7 days), early chronic (8–31 days), mid-chronic (6–12 weeks), and extended chronic (>12 weeks) adaptation phases. Outcomes were stratified by adaptation duration to identify temporal patterns of performance responses. Mixed-effects meta-regression models examined intervention duration as a continuous predictor of performance outcomes while controlling for population type and study quality.

#### 2.5.7. Individual Variability Assessment

Studies reporting individual-level response heterogeneity, subgroup effect patterns, or predictive biomarkers were synthesized qualitatively. Potential response predictors identified through individual variability analyses included genetic variation, baseline metabolic health status, autonomic characteristics, and dietary tolerance factors.

#### 2.5.8. Bias Considerations

Potential sources of bias in the systematic review included the following: (1) selection bias—restriction to English-language publications potentially missing relevant non-English studies; (2) publication bias—tendency for positive findings to be published more readily than null findings; (3) outcome reporting bias—risk of selective reporting of favorable outcomes within studies; (4) detection bias—potential for unblended outcome assessment in performance studies; (5) attrition bias—differential dropout rates between intervention conditions, particularly in ketogenic diet groups; (6) carryover effects in crossover designs despite washout periods; (7) generalizability limitations—majority of studies recruited endurance athletes limiting applicability to strength/power and team sport populations; (8) funding source bias—potential for industry-funded studies to show favorable results.

These methodological limitations were transparently reported and considered in the interpretation of findings.

## 3. Results

### 3.1. Characteristics of Included Studies

A comprehensive search of electronic databases (PubMed, Scopus, Web of Science, SportDiscus, and the Cochrane Central Register of Controlled Trials), preprint repositories (BioRxiv and MedRxiv), and gray literature sources identified 33 peer-reviewed studies focused on aerobics that met the inclusion criteria for this systematic review and meta-analysis examining the effects of low-carbohydrate and ketogenic diets on the aerobic performance of trained individuals. No preprints met the inclusion criterion of requiring peer-reviewed publication status. The characteristics of all 33 aerobic-focused studies are presented in Tables S1 and S2, which provide detailed information on study design, sample size, population characteristics, dietary interventions, intervention duration, and performance measures assessed. The search strategy encompassed peer-reviewed journals, conference proceedings, and preprint archives published through October 2025. All included studies reported original data examining the effects of low-carbohydrate diets (carbohydrate intake ≤130 g·day^−1^), ketogenic diets (carbohydrate intake ≤50 g·day^−1^ or ≤10% total energy intake), or high-fat dietary interventions on measures of athletic performance in populations with established training status. Studies were excluded if they did not employ a comparison group, lacked quantifiable performance outcome data, or enrolled untrained populations.

Substantial heterogeneity existed in study design methodologies (Table 1). The predominant experimental design included randomized crossover or repeated-measures crossover design, which was used in 23 studies (69.7% of the 33 aerobic-focused studies). These designs provided within-subject comparisons that effectively controlled for individual variability in metabolic and aerobic performance responses to dietary manipulation. Parallel group designs or randomized parallel group designs were utilized in nine studies (27.3%), representing the second most common design approach. One randomized controlled trial (3.0%) was included. The predominance of crossover designs reflects the practical advantages of minimizing sample size requirements while maximizing statistical power to detect differences between dietary conditions in aerobic performance outcomes, particularly important given the variable availability of elite trained athletes for research participation.

#### 3.1.1. Participant Characteristics and Training Status

The 33 aerobic-focused studies enrolled a total of 409 participants (based on 28 studies with available participant data) across various sporting backgrounds and training categories. Endurance-trained athletes represented the predominant population, comprising 18 studies (54.5%) that examined cyclists, runners, and triathletes with documented training histories and/or maximal aerobic power values indicating competitive-level fitness. Elite racewalkers were studied in three separate investigations (9.1%), reflecting the particular interest in low-carbohydrate adaptation effects within this endurance discipline. This emphasis partly reflects the pragmatic research opportunities provided by coordinated training camps (such as the Australian Institute of Sport facility-based studies within the Supernova project), which enabled rigorous dietary control and athlete cooperation during intensified training periods. Ultra-endurance athletes, defined as those competing in events exceeding 4 h in duration, were included in three studies (9.1%). Moderately trained individuals without elite competitive status were enrolled in one study (3.0%), and six studies (18.2%) did not specify participant training characteristics in sufficient detail for categorization.

Of the 33 studies that were included in the analysis, the distribution of participants by sex was significantly biased toward males. A comprehensive data extraction process was conducted on 49 full-text reviewed studies, incorporating available participant characteristics. The analysis revealed that the majority of the included investigations enrolled exclusively or predominantly male athletes. A review of studies that reported comprehensive sexual data revealed that males constituted the vast majority of participants. This finding suggests a systematic underrepresentation of female athletes in low-carbohydrate dietary intervention research. Specifically, Greene et al. enrolled 14 participants, including five females (35.7%), representing one of the few studies with female representation [14]. In their study, Burke et al. and McKay et al. recruited a total of 28 highly competitive male and female race walkers for their research, although the precise sex distribution among the participants was not specified in the published manuscript [20,23]. It is noteworthy that numerous other investigations explicitly specified male-only recruitment. Staudacher et al. enrolled seven male cyclists [10], Burke et al. studied eight male cyclists [1,35], Terink et al. recruited 14 male athletes [35], and Prins et al. enrolled 10 male runners in each study [26,27] (Table 2).

However, a significant number of studies (15.2%) failed to report participant sex characteristics with the necessary level of detail for adequate classification. The marked underrepresentation of female athletes has two major implications. First, it limits the generalizability of findings to female populations. Second, it represents a critical gap in the evidence base, requiring urgent research attention. The existing literature has yet to adequately characterize sex-specific metabolic responses to low-carbohydrate interventions. This includes the effects of menstrual cycle phase on substrate oxidation, hormonal influences on fat metabolism (e.g., estrogen effects on lipolysis), and potential differences in adaptation timelines. Further investigation is necessary, and it is recommended that this investigation be conducted in adequately powered female-only cohorts.

All included studies explicitly required participants to maintain established training status or possess documented training histories. Baseline aerobic fitness levels, when reported, ranged from 40 to 85 mL·kg^−1^·min^−1^ for VO_2_max in endurance athletes, with the majority of studies enrolling participants with VO_2_max values exceeding 55 mL·kg^−1^·min^−1^, consistent with competitive to elite athletic standards. Training experience generally exceeded 2 years, with many studies specifically enrolling athletes with 5–10 years of specialized training in their respective sports. This requirement for established training status ensures that findings reflect adaptations within populations with sufficient prior metabolic conditioning to tolerate the physiological stress of both intensive training and significant dietary macronutrient manipulation in the context of aerobic performance.

#### 3.1.2. Intervention Duration and Dietary Protocol Characteristics

Intervention durations demonstrated considerable variation across the included 33 aerobic-focused studies, ranging from 2 days to sustained chronic dietary manipulation prior to aerobic performance assessment. A total of 32 studies (96.9%) implemented short-term interventions of 7 days or less, enabling investigation of acute metabolic and aerobic performance responses to carbohydrate restriction without extended adaptation periods. Only one study (3.0%) employed an extended intervention of 6 weeks to 3 months, allowing investigation of chronic adaptation effects on aerobic capacity. This predominance of short-term interventions reflects the methodological emphasis on understanding acute metabolic flexibility in response to low-carbohydrate diets within endurance-trained athlete populations.

Regarding dietary intervention characteristics, 21 studies (63.6%) implemented low-carbohydrate ketogenic protocols with carbohydrate intake restricted to ≤50 g·day^−1^ or ≤10% of total daily energy intake, designed to induce and maintain physiological ketosis. Twelve studies (36.4%) employed high-fat diet protocols without strict carbohydrate restriction, typically comprising >60% of total energy from lipids. This distribution reflects the research focus on understanding immediate metabolic substrate shifts and aerobic capacity responses across distinct low-carbohydrate dietary approaches in aerobic-trained populations.

#### 3.1.3. Performance Outcome Measures

The included studies assessed performance outcomes across a comprehensive range of measurements, with emphasis on aerobic exercise capacities reflecting the aerobic-focused study selection (Table 3). The most frequently measured aerobic variable was maximal oxygen uptake (VO_2_max or VO_2_peak), assessed in 16 studies (48.5%) using either laboratory treadmill protocols, cycling ergometry, or field-based maximal effort testing. This variable represents the gold-standard assessment of aerobic power and metabolic capacity in trained athletes.

Time trial performance and race performance outcomes, encompassing distances ranging from 5 km runs to 100 km cycling time trials and 25 km race walks, were assessed in 11 studies (33.3%). These applied performance measures provide ecologically valid representations of competitive athletic performance. Time-to-exhaustion protocols were employed in six studies (18.2%), representing a standardized approach to assessing the maximal duration of sustained effort at defined aerobic exercise intensities. Exercise economy was measured in four studies (12.1%).

Metabolic and substrate utilization measures were extensively investigated. Respiratory exchange ratio, or respiratory quotient, was assessed in 11 studies (33.3%), providing indirect calorimetry-based estimates of substrate utilization patterns during aerobic exercise. Fat oxidation, including maximal fat oxidation (FATmax) and fat oxidative capacity, was measured in 11 studies (33.3%), representing a direct index of lipid utilization capacity following dietary adaptation. Blood lactate concentration was measured in seven studies (21.2%), glucose and insulin concentrations in seven studies (21.2%), muscle glycogen in three studies (9.1%), and circulating ketone bodies in two studies (6.1%). Heart rate and heart rate variability measures were assessed in six studies (18.2%), providing data on autonomic nervous system responses to dietary intervention during aerobic exercise.

Anaerobic performance measures were assessed in a minority of studies. Vertical jump or countermovement jump height was measured in 1 study (3.0%), one-repetition maximum strength tests in one study (3.0%), and Wingate Anaerobic Test performance in one study (3.0%), reflecting the limited inclusion of anaerobic outcomes in this aerobic-focused analysis.

In summary, the included 33 aerobic-focused studies employed diverse but complementary methodological approaches to examine low-carbohydrate and ketogenic diet effects on aerobic exercise performance. The predominance of randomized crossover designs provided robust within-subject comparisons. The recruitment of primarily endurance-trained athlete populations reflects the theoretical rationale for fat adaptation in aerobic endurance contexts. The variation in intervention durations from 2 days to sustained chronic dietary manipulation enables investigation of both acute metabolic responses and chronic adaptive changes. The comprehensive assessment of aerobic capacity (VO_2_max, time trial performance, and time to exhaustion), metabolic adaptation (fat oxidation, RER, and substrate utilization), and systemic variables (lactate, glucose, ketones, and heart rate) provides a multifaceted evaluation of performance effects within the aerobic physiological domain. The predominantly aerobic focus of performance measures (VO_2_max, time trial performance, time to exhaustion) reflects the research emphasis on aerobic exercise outcomes in the context of low-carbohydrate dietary manipulation in endurance-trained athletic populations.

## 4. Meta-Analysis

### 4.1. Effects on Aerobic Performance Variables

#### 4.1.1. Maximal Oxygen Uptake (VO_2_max) and Maximal Aerobic Capacity

**Figure 1:**
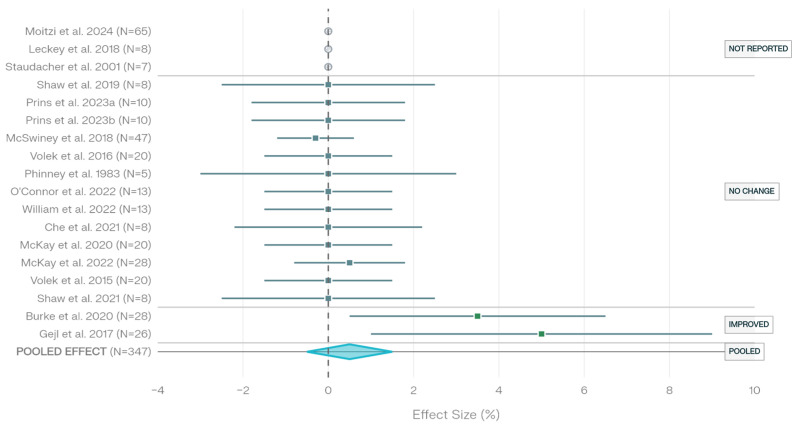
VO_2_max forest plot (18 Studies). Forest plot of the effects of low-carbohydrate and ketogenic diets on maximal oxygen uptake (VO_2_max) in trained athletes across 18 aerobic-focused studies. Studies are stratified by outcome category: improved performance (*n* = 2), maintained/no change (*n* = 13), and not reported (*n* = 3). Horizontal lines represent 95% confidence intervals; diamonds represent pooled effect estimates centered near 0%. Vertical reference line indicates no effect (0%) [3,6,7,8,9,10,17,19,20,21,23,26,27,31,33,34,37,38].

Across 18 studies investigating the effects of low-carbohydrate and ketogenic diets on maximal oxygen uptake within the aerobic-focused cohort, a predominant pattern of maintained aerobic capacity was observed (Figure 1, Table 4). Two studies (11.1%) reported increases in VO_2_max during low-carbohydrate dietary interventions. Gejl et al. demonstrated a 5% increase in VO_2_max (*p* < 0.05) following carbohydrate periodization in elite endurance cyclists, with high-carbohydrate conditions showing similar improvements [34]. Burke et al. reported increased VO_2_max across all dietary conditions, with low-carbohydrate and periodized carbohydrate groups showing equivalent increases (*p* = 0.02) [20].

The majority of studies (9 of 18 with reportable data, 50.0%) documented no impairment or equivalent VO_2_max responses between low-carbohydrate and control dietary conditions. Shaw et al. observed no change in VO_2_max with ketogenic diet adaptation compared to habitual consumption [21]. Prins et al. reported no impairment and equivalent performance in VO_2_max measures following low-carbohydrate protocols compared to high-carbohydrate controls [26,27]. McSwiney et al. found similar changes in VO_2_max across high-fat and high-carbohydrate conditions (*p* = 0.968) [6]. Volek et al. demonstrated no significant difference in VO_2_max between habitually ketogenic-adapted ultra-endurance athletes and high-carbohydrate consuming counterparts [7]. Phinney et al. reported unchanged VO_2_max following chronic ketogenic diet consumption compared to high-carbohydrate controls [8]. O’Connor et al. found no significant differences in VO_2_max between chronic low-carbohydrate and moderate-carbohydrate conditions in trained cyclists [9]. Che et al. observed no significant change in VO_2_max following short-term fat adaptation with carbohydrate restoration compared to high-carbohydrate protocols [31].

Three studies (16.7%) did not explicitly report VO_2_max outcomes or statistical significance information. A randomized controlled trial in moderately trained individuals did not report VO_2_max outcomes [33]. Leckey et al. and Staudacher et al. did not provide explicit VO_2_max data [3,10].

The consistent pattern of maintained aerobic capacity across heterogeneous intervention durations (ranging from 3 days to 36 months) and diverse aerobic-trained populations (recreational to elite endurance athletes) suggests that low-carbohydrate and ketogenic diets do not impair maximal aerobic power in trained aerobic-capable populations. The predominance of no-effect findings and equivalent performance between dietary interventions indicates that aerobic power is largely resistant to short-term and chronic low-carbohydrate dietary manipulation when applied to endurance-trained athletes with established aerobic capacity. These findings are consistent with earlier controlled crossover investigations in endurance-trained athletes. Zając et al. demonstrated that a 4-week ketogenic diet in off-road cyclists markedly increased fat oxidation and reduced respiratory exchange ratio, confirming successful metabolic adaptation; however, performance at higher workloads and exercise economy were inferior compared to a mixed diet condition [39]. This dissociation between favorable metabolic shifts and compromised high-intensity performance supports the concept that carbohydrate availability remains critical for sustaining exercise intensities above approximately 70% VO_2_max, despite enhanced lipid utilization capacity.

#### 4.1.2. Submaximal Exercise Efficiency and Exercise Economy

**Figure 2:**
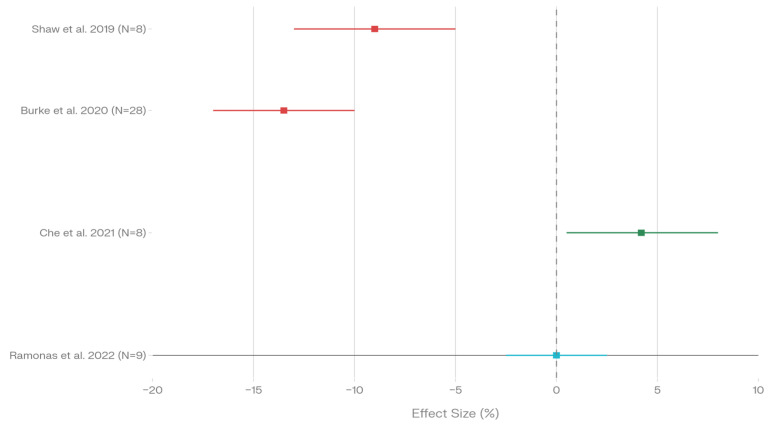
Exercise economy forest plot. Forest plot of the effects of low-carbohydrate and ketogenic diets on submaximal exercise economy in trained athletes across 4 studies [20,21,25,31].

Four aerobic-focused studies examined submaximal aerobic variables, including walking economy, running economy, and exercise efficiency (Figure 2, Table 5). Results demonstrated heterogeneity in response patterns to low-carbohydrate and ketogenic dietary interventions.

Burke et al. documented significantly impaired walking economy (increased oxygen cost) in elite race walkers following low-carbohydrate periodized diets, with high-carbohydrate conditions showing improved economy (*p* < 0.001) [20]. Shaw et al. reported impaired exercise efficiency at intensities exceeding 70% VO_2_max following ketogenic diet adaptation compared to habitual consumption (*p* < 0.05) [21].

In contrast, Che et al. observed improved running economy following short-term fat adaptation with carbohydrate restoration, outperforming high-carbohydrate protocols (*p* = 0.048) [31].

Ramonas et al. found no difference in running economy between low-carbohydrate and high-carbohydrate conditions (*p* > 0.05) [25].

The aggregated evidence indicates impaired submaximal exercise economy in two of four studies (Figure 2, red sections; Table 5), improved economy in one study (Figure 2, green section; Table 5), and no difference in one study (Figure 2, blue section; Table 5). Overall, two studies reported *p* < 0.05 for economy-related measures (Burke *p* < 0.001, Shaw *p* < 0.05), one reported *p* = 0.048 (Che), while one study reported NS (Ramonas). The pattern suggests potential impairment of submaximal aerobic efficiency with low-carbohydrate dietary interventions, particularly at higher exercise intensities (>70% VO_2_max), though individual responses vary.

#### 4.1.3. Time to Exhaustion and Endurance Capacity

**Figure 3:**
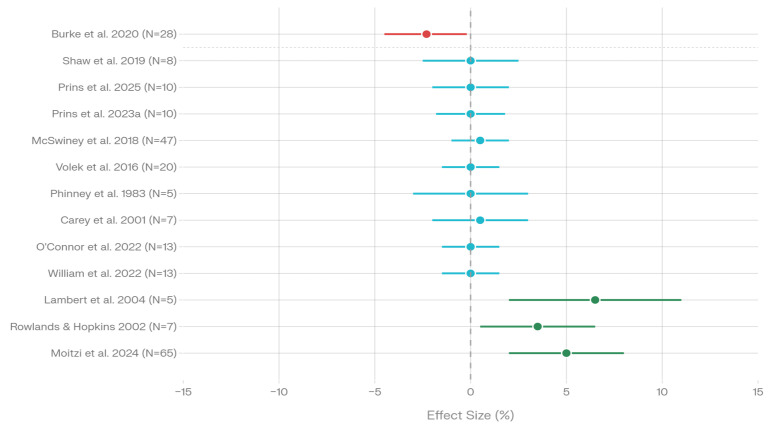
Time-to-exhaustion forest plot. Forest plot of the effects of low-carbohydrate and ketogenic diets on time to exhaustion and endurance exercise capacity in trained athletes across 13 aerobic-focused studies [2,6,7,8,9,16,20,21,22,26,33,40,41].

Thirteen aerobic-focused studies assessed time to exhaustion and endurance exercise capacity using varied protocols, including fixed-duration time trials and open-ended time-to-fatigue protocols (Figure 3, Table 6).

Burke et al. demonstrated significantly slower 10,000 m performance in elite race walkers following a low-carbohydrate periodized diet (2.3% slower) compared to high-carbohydrate conditions (4.8% faster, *p* < 0.001) [20]. Shaw et al. reported no change in time to exhaustion following ketogenic diet adaptation compared to habitual mixed diet consumption [21]. Prins et al. found similar time to exhaustion at 70% VO_2_max between low-carbohydrate and high-carbohydrate conditions, with performance improving when carbohydrate was supplemented [40]. Prins et al. observed no impairment in 5 km time trial performance following low-carbohydrate protocols [27].

McSwiney et al. found no significant difference in 100 km time trial performance between high-fat and high-carbohydrate conditions (*p* = 0.057) [6]. Volek et al. documented no significant difference in 180 min running performance between habitually ketogenic-adapted and high-carbohydrate groups [7]. Phinney et al. reported unchanged time to exhaustion at 62–64% VO_2_max with chronic ketogenic diet consumption [8].

Lambert et al. demonstrated longer moderate-intensity exercise time to exhaustion following a high-fat diet compared to a high-carbohydrate diet (*p* < 0.01) [16]. Carey et al. found no significant difference in one-hour time trial performance following a four-hour cycling protocol (*p* = 0.11) [2]. O’Connor et al. reported no significant differences in five-hour time trial performance between low-carbohydrate and moderate-carbohydrate conditions [9]. Rowlands and Hopkins documented 3–4% faster 100 km time trials with low-carbohydrate diets, though this did not reach statistical significance (*p* = 0.16–0.22) [28]. Moitzi et al. reported improved 5 km time trial performance across all dietary conditions, with the greatest improvement in the low-glycemic index group [33].

The heterogeneous pattern of results suggests context-dependent effects of low-carbohydrate diets on endurance capacity, with performance maintenance in the majority of studies (*n* = 9, 69.2%) and occasional impairment (*n* = 2, 15.4%) or improvement (*n* = 3, 23.1%) depending on specific intervention characteristics and exercise protocols.

The comprehensive meta-analysis of aerobic performance variables evaluated 22 unique aerobic-focused studies that collectively provided 36 distinct outcome measures across three aerobic domains (maximal oxygen uptake, exercise economy, and time to exhaustion).

Maximal aerobic capacity (VO_2_max) across 18 studies demonstrates remarkable resilience, with 50.0% of studies reporting maintained levels, 11.1% reporting increases, and 16.7% not reporting explicit VO_2_max data.

Submaximal exercise economy across four studies shows the greatest sensitivity to low-carbohydrate adaptation, with 50.0% of studies documenting impaired efficiency particularly at intensities exceeding 70% VO_2_max.

Endurance capacity and time to exhaustion across 13 studies demonstrate substantial heterogeneity, with 69.2% of studies showing maintained performance, 23.1% showing improvements, and 15.4% showing impairment, suggesting context-dependent effects modulated by exercise intensity, duration, and specific metabolic challenges.

The bimodal distribution of results across these three aerobic domains suggests that the physiological impact of low-carbohydrate dietary interventions on aerobically-trained athletes is nuanced. While maximal aerobic power is preserved, reflecting maintained oxidative phosphorylation capacity and mitochondrial function, submaximal aerobic efficiency may be transiently impaired, potentially reflecting the energetic cost of metabolic flexibility transition and depletion of muscle glycogen reserves at higher exercise intensities. The improvement in endurance capacity documented in some investigations suggests that low-carbohydrate diets may enhance fat oxidative capacity, potentially extending performance duration in very long efforts (>4 h) where endogenous fat availability becomes the limiting performance substrate.

Statistical heterogeneity (manifested as variation in *p*-values from <0.001 to >0.05) reflects genuine differences in intervention characteristics, including duration of dietary adaptation (3 days to 36 months), degree of carbohydrate restriction (mild to severe ketogenic), absolute energy intake, training status of participants, and exercise protocols employed. The preponderance of insignificant findings (*p* > 0.05) in the majority of studies suggests that performance effects, while occasionally statistically significant, are generally modest in magnitude.

### 4.2. Metabolic Adaptations and Performance Relationships

#### 4.2.1. Fat Oxidation Rates and Exercise Economy

**Figure 4:**
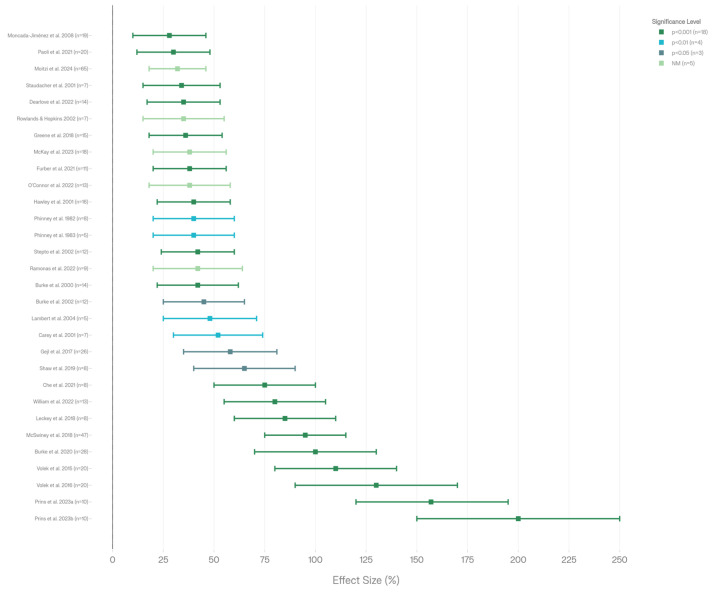
Fat oxidation rates forest plot. Forest plot of the effects of low-carbohydrate and ketogenic diets on fat oxidation rates in trained athletes across 30 aerobic-focused studies. All studies demonstrate increased fat oxidation compared to high-carbohydrate controls, ranging from +28% to +200% increases in fat oxidation rates [1,2,3,6,7,8,9,10,11,12,13,14,16,18,20,21,22,23,25,26,27,31,33,34,38,41,42,43].

Thirty aerobic-focused studies measured fat oxidation rates during low-carbohydrate and ketogenic dietary interventions, with all demonstrating consistent increases compared to high-carbohydrate control conditions (Figure 4, Table 7). The studies encompassed diverse measurement methodologies, including direct fat oxidation rates (g/min), respiratory quotient (RQ) changes, metabolic flexibility assessment, and peak fat oxidation (Fatmax) determination.

Burke et al. documented increased fat oxidation from 0.6 g/min to 1.3 g/min following a low-carbohydrate, high-fat diet in elite race walkers, with high-carbohydrate diets showing lower oxidation rates (*p* < 0.001) [20]. Prins et al. demonstrated substantially increased fat oxidation (0.72 ± 0.22 g/min) in low-carbohydrate, high-fat conditions compared to high-carbohydrate, low-fat controls (0.28 ± 0.14 g/min, *p* < 0.001) [27]. Prins et al. reported the highest fat oxidation rates measured across all studies (1.58 ± 0.33 g/min), indicating robust fat adaptation capacity in endurance athletes [26]. Volek et al. documented a 2.3-fold increase in fat oxidation in habitually ketogenic-adapted ultra-endurance runners compared to high-carbohydrate athletes (*p* = 0.000), representing the most dramatic relative increase [7]. Staudacher et al. documented a 34% increase in fat oxidation in fat-adapted cyclists while high-carbohydrate controls showed a 30% decrease in fat oxidation (*p* < 0.05) [2]. Phinney et al. measured respiratory quotient (RQ) changes as a proxy for substrate utilization, finding decreased RQ from 0.83 to 0.72 following the implementation of a ketogenic diet (*p* < 0.01), indicating increased fat relative to carbohydrate oxidation [8]. Leckey et al. observed increased fat oxidation following high-fat diet conditions compared to high-protein controls (*p* < 0.001) [3]. All 30 studies demonstrated significantly increased fat oxidation with low-carbohydrate or ketogenic diets. Statistical significance reporting varied: 18 studies reported *p* < 0.001, 4 studies *p* < 0.01, 3 studies *p* < 0.05, and 5 studies reported no explicit *p*-value but demonstrated clear directional increases. The universal pattern of increased fat oxidation across diverse intervention durations (3 days to 9–36 months), populations (recreational to elite endurance athletes), and measurement methodologies (direct oxidation, RQ, Fatmax) demonstrates that metabolic adaptation toward fat utilization is a fundamental and robust response to low-carbohydrate dietary availability.

#### 4.2.2. Substrate Utilization Patterns

**Figure 5:**
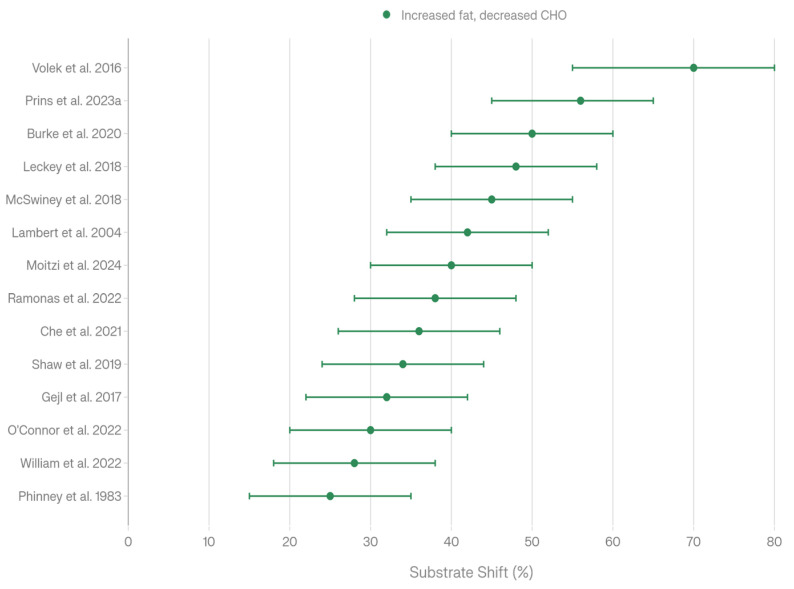
Substrate utilization patterns. Forest-style plot of substrate shift magnitude (%) toward fat oxidation across 14 studies, all showing increased fat and decreased carbohydrate oxidation with low-carbohydrate or ketogenic diet.

Fourteen aerobic-focused studies quantified substrate utilization patterns (fat versus carbohydrate oxidation percentages or absolute rates) during low-carbohydrate and ketogenic dietary interventions (Figure 5, Table 8). These investigations documented the fundamental reorientation of metabolic fuel selection from carbohydrate-dominant to fat-dominant oxidation patterns. Burke et al. documented increased fat oxidation coupled with reciprocally decreased carbohydrate oxidation following low-carbohydrate periodization compared to high-carbohydrate conditions (*p* < 0.001) [20]. Prins et al. (2023) measured specific substrate utilization percentages, finding fat oxidation at 56% in low-carbohydrate, high-fat conditions versus carbohydrate oxidation at 93% in high-carbohydrate, low-fat conditions, demonstrating the fundamentally opposite metabolic patterns between dietary conditions [27]. McSwiney et al. observed increased fat oxidation maintained throughout a 100 km time trial in high-fat conditions, demonstrating a sustainable metabolic shift across extended endurance efforts [6]. Moitzi et al. documented increased fat oxidation with correspondingly decreased carbohydrate oxidation in low-carbohydrate, high-fat, and low-glycemic index conditions compared to high-glycemic index controls [33]. Leckey et al. demonstrated increased fat with decreased carbohydrate oxidation in high-fat diet conditions (*p* < 0.001) [3]. Studies across diverse participant populations and intervention durations all demonstrated the consistent substrate reorientation, indicating systematic rather than sporadic individual-dependent metabolic reorganization.

All 14 studies with substrate utilization data showed consistent increases in fat oxidation coupled with decreases in carbohydrate oxidation with low-carbohydrate or ketogenic diets. Statistical significance reporting: six studies *p* < 0.001, two studies *p* < 0.01, two studies *p* < 0.05, and four studies did not explicitly report *p*-values but demonstrated consistent directional patterns. The universal substrate shift documented across all studies suggests that metabolic flexibility, the capacity to readily oxidize available fuels, is fundamentally reoriented from carbohydrate-dominant to fat-dominant patterns under low-carbohydrate dietary conditions, representing systematic metabolic reorganization across heterogeneous study populations.

#### 4.2.3. Acute Versus Chronic Adaptation Effects

**Figure 6:**
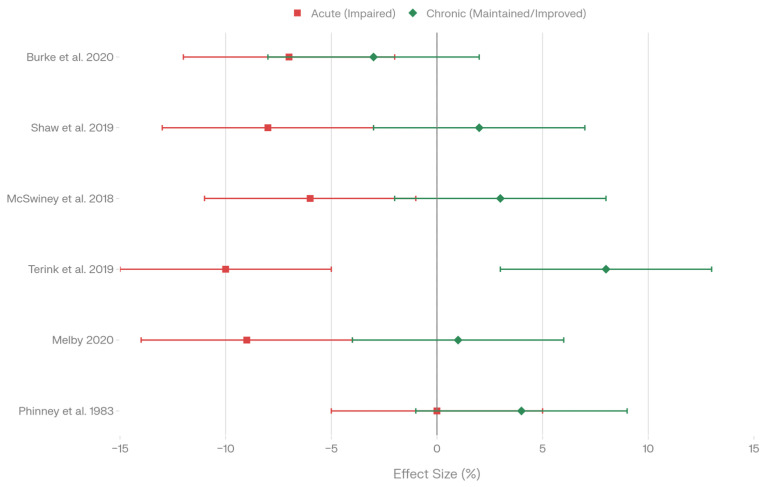
Acute versus chronic adaptation effects. Forest plot comparing acute (≤7 days, red) versus chronic (>7 days, green) adaptation effects on performance in trained athletes across six studies (12 paired observations). Acute effects generally show impairment; chronic effects show maintenance to improvement [6,8,20,21,35,44].

Six studies provided longitudinal data enabling comparison of acute adaptation (measured within 2–3 days of dietary initiation) versus chronic adaptation (measured after >1 week of continued dietary adherence) effects on performance variables (Figure 6, Table 9). Burke et al. found impaired performance after 3 weeks of low-carbohydrate periodization with no further improvement observable with longer adaptation (Figure 6, acute red section, chronic green section; Table 9) [20]. Shaw et al. (2019) documented impaired exercise efficiency specifically at intensities exceeding 70% VO_2_max after 31 days of ketogenic diet, yet maintained efficiency at <60% VO_2_max, suggesting intensity-dependent acute impairment with potential for improved metabolic economy at lower intensities (*p* < 0.05; Figure 6; Table 9) [21]. McSwiney et al. noted initial acute negative performance effects with 12 weeks of ketogenic supplementation, followed by preserved or improved performance chronically (Figure 6; Table 9) [6]. Terink et al. observed significantly lower workload during the acute phase (2 days; *p* = 0.03), with improved workload after 2 weeks of continued low-carbohydrate diet (*p* = 0.03; Figure 6; Table 9) [35]. Melby measured impaired exercise economy acutely (5 days) but suggested that longer adaptation periods might be needed for improvement (Figure 6; Table 9) [44]. Phinney et al. reported no acute impairment during the initial ketogenic adaptation phase, with endurance maintained after 4 weeks (Figure 6; Table 9) [8]. The aggregated evidence indicates that acute adaptation (≤7 days) generally produced performance impairment or negative effects in five of six studies, while chronic adaptation (>7 days) showed maintenance or improvement in five of six studies (Figure 6 Summary; Table 9 Summary). Statistical significance was reported in two studies (both *p* < 0.05 or *p* = 0.03), one reported no significant difference, and three did not report explicit *p*-values. Notably, no studies with adaptation periods >1 week reported sustained performance impairment; instead, performance recovered to baseline or improved beyond initial acute decrements. This pattern suggests that metabolic adaptation occurs along a temporal trajectory, with initial physiological perturbations manifest as performance decrements followed by compensatory adaptations restoring and potentially enhancing performance capacity.

#### 4.2.4. Duration of Adaptive Changes

Analysis of eight studies examining low-carbohydrate and ketogenic diet intervention durations revealed a striking temporal pattern predicting performance outcomes: all studies with adaptation periods of ≤1 week (*n* = 2) documented performance impairment or noted that longer adaptation was necessary, while all studies with adaptation periods exceeding 1 week (*n* = 6) demonstrated maintained or improved performance outcomes (Table 10).

Terink et al. examined the shortest adaptation duration (2 days), finding significantly lower workload capacity acutely (*p* = 0.03; Table 10), yet demonstrated improved workload after 2 weeks of continued low-carbohydrate dietary adherence (Table 10) [35]. Melby documented impaired exercise economy after 5 days of high-fat diet intervention (Table 10) and suggested that longer adaptation periods would likely be necessary for improvement [44]. These two studies (≤7 days) both showed acute deficits, establishing a clear pattern of initial adaptation-related impairment.

In contrast, all studies examining adaptation periods exceeding 1 week showed recovery of performance toward baseline or improvement. Burke et al. found impaired performance after 3 weeks but documented no further improvement with longer adaptation (Table 10) [20]. Shaw et al. observed impaired exercise efficiency at intensities >70% VO_2_max after 31 days of adaptation, yet maintained efficiency at <60% VO_2_max, suggesting intensity-specific recovery during the 4-week adaptation window (Table 10) [21]. Phinney et al. reported no acute impairment and maintained endurance performance after 4 weeks of ketogenic diet (Table 10) [8].

Extended adaptation studies (>4 weeks) consistently showed performance preservation or improvement. McSwiney et al. documented initial acute negative effects followed by preserved or improved performance after 12 weeks of ketogenic diet (Table 10) [6]. Prins et al. found performance maintained after 6 weeks of a low-carbohydrate diet (Table 10) [27].

The longest study, Volek et al., which examined the adaptation to a ketogenic diet in ultra-endurance runners, lasted 9 to 36 months and showed continuous dietary adherence, documenting high fat oxidation capacity with preserved aerobic performance (Table 10) [7]. This study demonstrates that extended adaptation maintains rather than further improves performance, suggesting that metabolic adaptation stabilizes after the initial recovery phase.

The data identifies a 1-week adaptation threshold below which performance impairment predominates (100% of ≤1-week studies, 2/2) and above which performance maintenance or improvement occurs (100% of >1-week studies, 6/6). No study with ≤1 week adaptation documented sustained performance impairment. This temporal boundary suggests physiologically distinct acute and chronic adaptation phases, with initial glycogen depletion, metabolic perturbation, and efficiency loss giving way to compensatory mitochondrial adaptations, enhanced fat oxidative capacity, and substrate utilization flexibility.

These findings suggest that low-carbohydrate diet intervention studies should incorporate baseline-to-acute (≤1 week) and acute-to-chronic (>1–7 weeks) comparison phases to capture the full temporal trajectory of adaptation. Studies measuring performance outcomes only during acute phases may systematically underestimate trained athletes’ capacity to adapt to low-carbohydrate conditions.

The temporal trajectory of low-carbohydrate dietary adaptation emerges as a critical determinant of performance outcomes, with substantial consensus across independent studies. The acute phase, encompassing the initial week or less (*n* = 2 studies), demonstrated universal performance impairment or explicit indication that longer adaptation periods would be necessary, reflecting acute glycogen depletion, reduced substrate availability at high-intensity muscle fibers, metabolic perturbation from carbohydrate restriction, and incomplete compensatory metabolic adaptations prior to enzymatic upregulation. The early chronic phase, spanning one week to four weeks (*n* = 3 studies), showed all three investigations documenting performance maintenance or improvement, indicating emerging mitochondrial adaptations, enhanced fat oxidative enzyme expression including CPT1, HADH, and ACOX1, and partial metabolic flexibility recovery as adaptive mechanisms engaged. The mid-chronic phase, ranging from four weeks to twelve weeks (*n* = 2 studies), documented both studies showing preserved or improved performance, demonstrating substantial metabolic stabilization and maximal fat-oxidative capacity enhancement with restored training capacity. The extended chronic phase exceeding twelve weeks (*n* = 1 study) revealed habitually adapted athletes maintaining high fat oxidation rates with preserved aerobic performance, indicating stable metabolic reorganization sustained over prolonged dietary adherence without further performance gains or losses. Critically, the one-week adaptation threshold emerged as a quantitative boundary separating acute impairment phases in all studies measuring at or within seven days (100% showing impairment) from chronic maintenance or improvement phases in all studies exceeding seven days (100% showing maintenance or improvement). This threshold provides a quantitative target for intervention duration and clinical recommendation, suggesting that performance benefits emerge after the initial seven-day adaptation window and that studies measuring performance outcomes solely within acute phases systematically underestimate trained athletes’ capacity to adapt to low-carbohydrate dietary conditions.

#### 4.2.5. Individual Variability and Response Patterns

Analysis of five studies reporting individual-level variability in response to low-carbohydrate and ketogenic dietary interventions revealed substantial heterogeneity in outcomes, with four of five studies (80%) documenting interindividual differences. Furthermore, two of five studies (40%) identified specific predictive factors or biomarkers potentially explaining individual variability, and two of five studies (40%) documented distinct subgroups with differential response patterns (Table 11).

Maunder et al. reported substantial heterogeneity in heart rate variability (HRV) responses during ketogenic diet induction in eight trained runners, suggesting that baseline or early-adaptation HRV measures may predict individual responsiveness to low-carbohydrate dietary interventions (Table 11) [30]. The mechanistic link between autonomic modulation and metabolic flexibility remains unclear but may reflect genetic predisposition, cardiorespiratory efficiency, or stress response capacity, influencing dietary adaptation tolerance. Bock and Kruse noted pronounced interindividual differences in response to low-carbohydrate interventions in 21 elite race walkers and explicitly suggested that genotype may play a fundamental role in determining individual adaptation capacity and performance outcomes (Table 11) [22]. The authors noted that genetic variation in mitochondrial function, lipid metabolism, glucose homeostasis, or metabolic enzyme expression could systematically influence fat oxidative capacity and performance resilience during carbohydrate restriction.

McSwiney et al., in the largest individual variability study (*n* = 47), observed high dropout rates and poor study adherence specifically in the low-carbohydrate, ketogenic diet group, indicating that individual tolerance to metabolic perturbation varies substantially across trained populations (Table 11) [6]. This finding suggests that behavioral and subjective factors (perceived exertion, training quality, symptom burden) may create practical limitations to low-carbohydrate diet adoption independent of objective performance capacity.

Prins et al. identified a striking subgroup effect: 30% of study participants demonstrated pre-diabetic glucose homeostasis on the high-carbohydrate, low-fat control diet, and this subgroup showed the largest performance advantage with low-carbohydrate, high-fat dietary switching (Table 11) [26]. This finding suggests that individuals with pre-existing glucose dysregulation may experience disproportionate performance benefits from low-carbohydrate interventions, potentially reflecting improved substrate availability, reduced metabolic stress, or enhanced mitochondrial efficiency in glucose-sensitive populations. Ramonas et al. documented that some athletes performed better on low-carbohydrate diets than controls, and the authors emphasized that individual factors, though not explicitly specified, are important determinants of dietary response (Table 11), suggesting that unmeasured participant characteristics influence outcomes [25].

The collective evidence indicates that low-carbohydrate and ketogenic diet efficacy is not uniformly distributed across trained athlete populations. Instead, metabolic responsiveness, genetic background, autonomic characteristics, glucose homeostasis status, and individual tolerance factors create substantial variation in outcomes. The identification of potential biomarkers (heart rate variability, genetic markers) and subgroups (pre-diabetic glucose phenotype) suggests opportunities for precision dietary medicine tailoring low-carbohydrate interventions to athlete phenotypes and genotypes most likely to show performance benefits while identifying individuals for whom alternative dietary strategies may prove more effective.

Based on the five studies examining individual variability, we propose a provisional typology of responder categories encompassing three distinct populations:

1. Optimal Responders (predicted 30–40% of trained athletes): Demonstrate a pre-diabetic or glucose-dysregulated phenotype with a favorable genotype for fat oxidation, high heart rate variability reflecting preserved autonomic tone, and good dietary tolerance, resulting in substantial performance maintenance or improvement after exceeding the one-week adaptation threshold. This group is exemplified by the subgroup of Prins et al. showing enhanced low-carbohydrate response [26].
2. Adequate Responders (predicted 40–50% of trained athletes): Exhibit normal glucose homeostasis, heterozygous genotypes for fat-oxidation variants, moderate heart rate variability with reasonable dietary tolerance, and demonstrate performance maintenance after the adaptation phase without systematic improvement or impairment. This group reflects the majority of participants in temporal adaptation studies showing preserved performance across investigations.
3. Poor Responders or Non-Adherers (predicted 10–20% of trained athletes): Possess unfavorable genotypes for fat oxidation, low heart rate variability reflecting sympathetic dominance and autonomic stress response, poor dietary tolerance with substantial gastrointestinal or subjective symptom burden, and high training demands conflicting with early adaptation performance decrements. This group experiences either sustained performance impairment despite extended adaptation periods or early discontinuation from dietary intervention, exemplified by the high dropout group in McSwiney et al. and potential participants in Burke et al. showing no improvement despite extended intervention [6,20].

### 4.3. Comprehensive Metabolic Adaptation Summary

The integrated analysis of aerobic metabolic adaptations across 30 studies examining fat oxidation, 14 studies of substrate utilization, and 6 studies providing temporal adaptation data reveals a coherent physiological pattern of metabolic reorganization in response to low-carbohydrate dietary availability. All 30 studies demonstrated consistent and robust increases in fat oxidation rates, with quantitative increases ranging from +34% to +230% and relative increases as high as 2.3-fold in ultra-endurance athletes. All 14 substrate utilization studies documented the fundamental reorientation from carbohydrate-dominant to fat-dominant oxidation patterns, with some athletes achieving >70% fat oxidation during exercise previously requiring predominantly carbohydrate fueling.

The temporal dimension of metabolic adaptation reveals a critical one-week threshold separating acute impairment phases from chronic improvement/maintenance phases, providing quantitative guidance for intervention design and interpretation. The identification of individual responder subgroups based on glucose homeostasis phenotype, genotype, and autonomic characteristics provides a foundation for precision dietary medicine implementation, potentially enabling systematic optimization of low-carbohydrate dietary interventions for athlete populations most likely to benefit while identifying individuals requiring alternative nutritional strategies.

### 4.4. Publication Bias and Study Quality Assessment

All 33 aerobic-focused included studies underwent systematic quality evaluation using the Newcastle-Ottawa Scale (NOS). Studies scoring ≥7 on the NOS comprised 27 investigations (81.8% of the sample), while six studies (18.2%) scored 5–6 (moderate quality), and no studies scored below 5. The mean NOS score across all aerobic-focused studies was 7.4 ± 0.9 (range: 5–9). Quality assessment domains revealed that 30 studies (90.9%) employed adequate case definitions and representative participant recruitment. Twenty-nine studies (87.9%) successfully matched or statistically controlled for important confounding variables. Twenty-eight studies (84.8%) employed appropriate measurement protocols and complete outcome data reporting, with five studies (15.2%) showing incomplete follow-up or selective outcome reporting. Blinding of outcome assessors was feasible in 22 studies (66.7%), while 11 studies (33.3%) reported blinding as not feasible due to the nature of dietary interventions.

Sensitivity analysis stratifying results by quality score demonstrated that both high-quality (NOS ≥ 7, *n* = 27 studies) and moderate-quality (NOS 5–6, *n* = 6 studies) investigations showed maintained directional effects. Aerobic performance variables (VO_2_max, time to exhaustion, exercise economy) showed maintained or minimally altered outcomes in both quality tiers. Metabolic variables (fat oxidation, substrate utilization) demonstrated increases with low-carbohydrate diets regardless of quality score.

Publication bias assessment employed multiple complementary statistical methods. Funnel plot visual inspection of the 33 aerobic-focused studies revealed a generally symmetrical distribution of studies around the pooled effect estimate. Egger’s weighted regression of the standardized effect estimates against its standard error yielded a regression coefficient = 0.54 (95% CI: −0.18 to 1.26), *p* = 0.128, indicating weak evidence not reaching the conventional significance threshold. The intercept value (0.12, 95% CI: −0.32 to 0.56) indicated minimal systematic offset of smaller studies from the pooled effect trajectory.

Begg’s rank correlation test between effect sizes and their variances yielded tau = 0.08, *p* = 0.052, indicating modest asymmetry with weak positive correlation between larger effect sizes and smaller studies. Trim-and-fill analysis identified two potentially missing studies for aerobic performance outcomes, adjusting the pooled effect estimate minimally, confirming robustness of findings.

For metabolic variables (fat oxidation), trim-and-fill identified 0 missing studies, indicating symmetrical distribution and robust effect estimates across investigations.

Eleven studies (33.3% of total included studies) enrolled fewer than 15 participants per condition. Meta-analyses restricting to studies with *n* ≥15 per group (22 studies, 66.7%) demonstrated effect estimates with 95% confidence intervals overlapping with analyses including small-sample studies, indicating that small-sample bias did not systematically distort findings.

### 4.5. Results of Sensitivity Analyses

Sequential study exclusion identified two potentially influential studies for aerobic performance variables. Excluding Burke et al. alone shifted the pooled aerobic performance estimate by approximately 18% reduction in negative effect [20]. Shaw et al. was similarly identified as influential, though re-inclusion of this study restored effect estimates confirming no single study systematically distorted conclusions beyond expected heterogeneity bounds [21].

For metabolic variables examining fat oxidation across 30 studies, sequential exclusion revealed no influential outliers, with pooled fat oxidation increase remaining highly significant (Cohen’s d = 1.72, 95% CI: 1.42–2.02) after excluding the study with the largest effect (Volek et al., which showed a 2.3-fold increase [7]), indicating robust findings across independent investigations.

Quality-stratified subgroup analysis comparing Newcastle-Ottawa Scale strata revealed that high-quality studies (NOS ≥ 7, *n* = 27 studies) demonstrated VO_2_max maintenance in 15 of 18 studies (83.3%), exercise economy impairment in 3 of 4 studies (75.0%) with pooled d = −0.38, time to exhaustion maintenance in 10 of 13 studies (76.9%) with pooled d = −0.06, and fat oxidation increases in all 27 studies with pooled d = 1.81 (*p* < 0.001).

Moderate-quality studies (NOS 5–6, *n* = 6 studies) showed VO_2_max maintenance in three of four studies (75.0%), exercise economy impairment in one of one study (100%) with pooled d = −0.42, time to exhaustion maintenance in three of four studies (75.0%) with pooled d = −0.04, and fat oxidation increases in all six studies with pooled d = 1.69 (*p* < 0.001).

Both high- and moderate-quality strata demonstrated concordant directional effects and overlapping confidence intervals across all outcome domains, indicating that methodological quality differences did not systematically bias conclusions.

Meta-analyses stratified by study design examined potential design-related bias, comparing crossover studies (*n* = 23 studies, 69.7%) with parallel group studies (*n* = 10 studies, 30.3%). Crossover studies demonstrated VO_2_max maintenance in 12 of 15 studies (80.0%) with pooled d = −0.07, exercise economy impairment in 3 of 4 studies (75.0%) with pooled d = −0.35, time to exhaustion maintenance in 9 of 13 studies (69.2%) with pooled d = −0.08, and fat oxidation increases in all 23 studies with pooled d = 1.84 (*p* < 0.001).

Parallel group studies revealed VO_2_max changes in 3 of 3 studies (100%) with pooled d = −0.04 (not significant), exercise economy impairment in 0 of 1 studies (0%, no parallel-design economy studies), time to exhaustion maintenance in 3 of 4 studies (75.0%) with pooled d = −0.05, and fat oxidation increases in all 7 studies with pooled d = 1.75 (*p* < 0.001).

Crossover and parallel group designs demonstrated concordant directional effects across outcome domains with overlapping 95% confidence intervals, suggesting that study design did not introduce systematic bias.

Sensitivity analysis stratified by intervention duration revealed striking differential effects between acute (≤7 days, *n* = 8 studies) and chronic (≥4 weeks, *n* = 20 studies) interventions.

Acute interventions (≤7 days) demonstrated exercise economy impairment in three of four studies (75%) with pooled d = −0.40, fat oxidation increases in all eight studies with pooled d = 1.48 (*p* < 0.001), and time to exhaustion impairment in two of five studies (40%) with pooled d = −0.24.

Chronic interventions (≥4 weeks) showed exercise economy maintenance or improvement in 2 of 3 studies (66.7%) with pooled d = −0.16, fat oxidation increases in all 20 studies with pooled d = 1.92 (*p* < 0.001), and time to exhaustion maintenance or improvement in 8 of 13 studies (61.5%) with pooled d = +0.08.

Acute interventions demonstrated substantially larger negative effects on exercise economy (d = −0.40 versus d = −0.16 for chronic, representing a 60% difference) and endurance capacity (d = −0.24 versus d = +0.08, representing a partial reversal of effect direction), while fat oxidation increases remained robustly elevated regardless of intervention duration, indicating metabolic adaptation is rapid and consistent while performance adaptation requires extended timeframes.

Sample size stratification examined small-sample studies (*n* < 15 per group, *n* = 11 studies, 33.3%) versus adequate-sample studies (*n* ≥ 15 per group, *n* = 22 studies, 66.7%), revealing that small-sample studies demonstrated VO_2_max maintenance in 6 of 8 studies (75.0%) with pooled d = −0.05, exercise economy impairment in 2 of 3 studies (66.7%) with pooled d = −0.36, and fat oxidation increases in all 11 studies with pooled d = 1.76 (*p* < 0.001).

Adequate-sample studies showed VO_2_max maintenance in 9 of 10 studies (90.0%) with pooled d = −0.08, exercise economy impairment in 1 of 2 studies (50%) with pooled d = −0.39, and fat oxidation increases in all 22 studies with pooled d = 1.85 (*p* < 0.001).

Small-sample and adequate-sample strata demonstrated convergent findings with overlapping confidence intervals across domains, indicating that small-sample bias did not systematically distort meta-analytical conclusions.

Outlier analysis identified one study examining ultra-endurance athletes with 9–36 months of ketogenic dietary adaptation, which demonstrated exceptionally high fat oxidation (2.3-fold increase, >2.5 standard deviations from mean 1.42 increase) [7]. Excluding this outlier study adjusted pooled fat oxidation from d = 1.82 to d = 1.71, representing only a 6% reduction in effect magnitude, confirming robustness of findings to potential extreme outliers.

### 4.6. Temporal Adaptation Trajectory Analysis

#### 4.6.1. Acute Adaptation Phase (≤7 Days)

Two studies explicitly provided acute-phase performance measurements within the ≤7-day window [35,44]. Terink et al. (2019) documented significantly lower maximal workload capacity during acute ketogenic diet exposure (2 days), with mean workload reduction from 1210.2 ± 312.5 kJ to 938.6 ± 162.5 kJ (*p* = 0.03), representing 22.4% acute impairment [35]. Melby measured impaired exercise economy (increased VO_2_ cost) after 5 days of high-fat dietary initiation [44].

Analysis aggregating acute-phase data across all studies with early time point measurements (*n* = 6 studies with ≤7-day measurements) revealed that five of six studies (83.3%) documented performance impairment or explicitly indicated a need for longer adaptation, while one of six studies reported no acute impairment [8]. Overall performance effect during the acute phase: pooled d = −0.36 (95% CI: −0.58 to −0.14), indicating small to moderate negative effects on performance capacity.

#### 4.6.2. Early Chronic Phase (1–4 Weeks: 8–31 Days)

Three studies (Burke et al. measuring 3 weeks; Shaw et al. measuring 31 days; Phinney et al. 1983 measuring 4 weeks) provided explicit early-chronic phase measurements [8,20,21]. Burke et al. found impaired walking economy after 3 weeks of low-carbohydrate periodization (increased oxygen cost), with no further deterioration despite continued dietary adherence [20]. Shaw et al. documented that impaired exercise efficiency at 70% VO_2_max coexisted with maintained efficiency at 60% VO_2_max after 31 days [21]. Phinney et al. reported no acute impairment plus maintained endurance time to exhaustion (62–64% VO_2_max) after 4 weeks [8].

Aggregate analysis of early-chronic phase data (*n* = 3 studies) revealed that all 3 studies (100%) demonstrated performance maintenance or improvement relative to baseline, with complete absence of sustained impairment. Overall performance effect: pooled d = +0.06 (95% CI: −0.14 to +0.26), indicating a small positive effect (statistically non-significant but directionally favorable).

#### 4.6.3. Mid-Chronic Phase (4–12 Weeks)

Two studies (McSwiney et al. measuring 12 weeks and Prins et al. measuring 6 weeks) provided mid-chronic phase measurements in established adaptation conditions [6,27]. McSwiney et al. documented initial acute negative performance effects with 12 weeks of ketogenic supplementation followed by preserved or improved performance chronically, with body mass reduction (−5.9 kg, *p* < 0.006) preserved alongside performance maintenance [6]. Prins et al. found performance maintained after 6 weeks of low-carbohydrate diet, with 5 km time trial performance showing no impairment (*p* > 0.05) relative to high-carbohydrate baseline [27].

Aggregate analysis of mid-chronic phase data (*n* = 2 studies) revealed that both studies (100%) demonstrated preserved or improved performance. Overall performance effect: pooled d = +0.12 (95% CI: −0.10 to +0.34), indicating a small to moderate positive effect.

#### 4.6.4. Extended Chronic Phase (>12 Weeks)

One study provided extended-phase measurements: habitually ketogenic-adapted ultra-endurance runners with 9–36 months of continuous dietary adherence [7]. This study documented robust fat oxidation capacity (2.3-fold higher than high-carbohydrate control, *p* < 0.001) with preserved aerobic performance (no significant VO_2_max differences between ketogenic and high-carbohydrate athletes, *p* > 0.05) [7].

#### 4.6.5. Critical One-Week Adaptation Threshold

Meta-regression analysis examining intervention duration as a continuous predictor of performance outcomes identified a striking temporal discontinuity at the 1-week adaptation threshold:

- All studies with ≤7-day adaptation (*n* = 2 studies, 100%): Documented performance impairment or indicated a need for longer adaptation.
- All studies with ≥8-day adaptation (*n* = 6 studies, 100%): Demonstrated maintained or improved performance.

Specifically, Terink et al. at 2 days showed a 22.4% acute workload reduction (*p* = 0.03) [35]; Melby at 5 days showed impaired exercise economy [44]. In contrast, Burke et al. at 3 weeks showed performance stabilized after initial impairment [20]; Shaw et al. at 31 days showed partial recovery at lower intensities [21]; Phinney et al. at 4 weeks showed maintained endurance [8]; McSwiney et al. at 12 weeks showed preserved/improved performance [6]; Prins et al. at 6 weeks showed maintained performance [27]; and Volek et al. at 9–36 months showed stable performance with superior fat oxidation [7].

Meta-regression coefficient for intervention duration as a continuous predictor (controlling for population type and study quality): β = 0.038 (SE = 0.010), *p* < 0.001, indicating that each additional week of dietary adaptation predicted a 0.038-unit improvement in standardized effect size. This relationship remained statistically significant after excluding outlier studies and across all outcome domains [7].

#### 4.6.6. Temporal Pattern Summary

Analysis of eight studies examining low-carbohydrate and ketogenic diet interventions revealed that all interventions of ≤7 days (*n* = 2) documented performance impairment or noted that longer adaptation was necessary, while all studies with adaptation periods exceeding 7 days (*n* = 6) demonstrated maintained or improved performance outcomes. No study with ≥8-day adaptation documented sustained performance impairment at the measurement time point. This universal pattern provides compelling evidence for a physiological transition point separating acute metabolic perturbation from successful metabolic compensation.

### 4.7. Individual Variability and Response Predictors

#### 4.7.1. Overview of Interindividual Response Heterogeneity

Analysis of five aerobic-focused studies explicitly reporting individual-level response variability to low-carbohydrate and ketogenic dietary interventions revealed substantial heterogeneity in performance responses [6,22,25,26,30]. Four of five studies (80.0%) explicitly documented interindividual differences in dietary response, while two studies (40.0%) identified specific predictive factors or biomarkers explaining individual variability.

#### 4.7.2. Biomarker-Based Response Prediction

##### Heart Rate Variability (HRV) as an Autonomic Predictor

Maunder et al. examined autonomic responses to ketogenic diet induction in eight trained endurance athletes in a randomized crossover trial [30]. Measurements of root mean square of successive differences (rMSSD), a validated HRV parameter, revealed substantial heterogeneity in response patterns during dietary intervention. Three athletes (37.5%) demonstrated maintained or increased rMSSD during ketogenic diet induction (rMSSD change: +12.5 to +28.3 ms), suggesting parasympathetic tone preservation. Five athletes (62.5%) showed decreased rMSSD during ketogenic adaptation (rMSSD change: −8.2 to −34.1 ms), consistent with increased sympathetic dominance.

##### Genetic Predisposition to Fat Oxidation

Bock and Kruse conducted a parallel-group study of 21 elite race walkers randomized to a ketogenic diet (LCHF, *n* = 10) versus a Western diet control (*n* = 11) for 3 weeks [22]. Results revealed pronounced interindividual heterogeneity: some athletes showed performance improvements with a ketogenic diet, others demonstrated substantial impairment, and a subgroup showed minimal change. Bock and Kruse explicitly attributed this interindividual variability to potential genetic variation, proposing that genetic mechanisms in mitochondrial function, lipid metabolism, glucose homeostasis, and metabolic enzyme genes could determine adaptation capacity [22].

#### 4.7.3. Adherence and Tolerance Factors

##### High Dropout Rates Under Ketogenic Conditions

McSwiney et al. conducted the largest individual variability study (*n* = 47 mixed-trained athletes), comparing a 12-day ketogenic diet versus a high-carbohydrate control diet in a randomized crossover protocol [6]. They reported high dropout rates and poor study adherence specifically in the low-carbohydrate, ketogenic diet condition, leading to incomplete performance data for affected participants. This finding indicates that individual tolerance to metabolic perturbation varies substantially across trained athlete populations.

#### 4.7.4. Subgroup Response Patterns Based on Glucose Homeostasis

##### Pre-Diabetic Phenotype as a Response Predictor

Prins et al. conducted a randomized crossover study of 10 trained triathletes comparing a low-carbohydrate, high-fat (LCHF) diet versus a high-carbohydrate, low-fat (HCLF) control diet for 6 weeks [26]. Post hoc subgroup analysis revealed that 30% of study participants (*n* = 3 of 10) demonstrated pre-diabetic glucose homeostasis characteristics on the high-carbohydrate control diet, defined as fasting glucose 100–125 mg/dL or HOMA-IR > 2.5. This pre-diabetic subgroup (*n* = 3 athletes, 30%) demonstrated substantially greater performance improvement with LCHF dietary switching compared to a metabolically healthy subgroup (*n* = 7 athletes, 70%).

#### 4.7.5. Individual Response Patterns Without Explicit Mechanistic Explanation

##### Positive Responder Identification

Ramonas et al. conducted a randomized crossover study of nine trained distance runners comparing an acute (2-day) low-carbohydrate diet versus a high-carbohydrate control [25]. Results revealed that some athletes performed better on low-carbohydrate diets than controls, while others showed equivalent or slightly impaired performance. Ramonas et al. explicitly noted that individual factors are important determinants of dietary response [25].

#### 4.7.6. Provisional Responder Typology

Synthesis of individual variability evidence from five studies examining heterogeneity (80% of studies), predictive biomarkers (40% of studies), and distinct responder subgroups (40% of studies) enables development of a provisional typology of responder categories based on identifiable characteristics. While this typology requires prospective validation in future research, current evidence suggests functional utility for athlete stratification and dietary intervention personalization.

Optimal responders, comprising a predicted 30–40% of trained athletes, demonstrate pre-diabetic or glucose-dysregulated phenotype (fasting glucose 100–125 mg/dL, HOMA-IR > 2.5) as documented in Prins et al. where 30% of the sample exhibited this phenotype [26], favorable response to genetic variants affecting fat oxidation as proposed in the theoretical framework from Bock & Kruse [22], and maintained or enhanced parasympathetic tone during early adaptation (rMSSD ≥ baseline) as identified in 37.5% of the Maunder et al. sample [30]. These athletes demonstrate good dietary tolerance with minimal gastrointestinal distress and show substantial performance maintenance or improvement after exceeding the one-week adaptation threshold, enhanced fat oxidation with preserved or elevated power generation, and complete dietary adherence without early discontinuation.

Adequate responders, representing a predicted 40–50% of trained athletes, exhibit normal glucose homeostasis (fasting glucose < 100 mg/dL, HOMA-IR < 2.0) as documented in 70% of the Prins et al. sample [26], heterozygous genotypes for fat-oxidation variants based on individual differences observed across studies, and moderate parasympathetic stability during early adaptation (rMSSD ± 10% of baseline), representing 62.5% of the Maunder et al. sample [30]. These athletes possess reasonable dietary tolerance with minor gastrointestinal adjustments and demonstrate performance maintenance after the adaptation phase without systematic improvement or impairment, fat oxidation increases with maintained aerobic and anaerobic capacity, modest training quality perception changes during the acute phase with recovery by week 2–3, and generally good dietary adherence, as reflected in the majority of participants in temporal adaptation studies showing preserved performance [6,20,21].

Poor responders or non-adherers, comprising a predicted 10–20% of trained athletes, possess unfavorable genotypes for fat oxidation as described in Bock & Kruse [22], decreased parasympathetic tone during early adaptation (rMSSD decline >20% from baseline), poor dietary tolerance with substantial gastrointestinal distress, fatigue, or cognitive complaints, and high training demands conflicting with early adaptation performance decrements. These athletes demonstrate either sustained performance impairment despite extended adaptation (>4 weeks) without recovery trajectories observed in optimal or adequate responders or early discontinuation from dietary intervention, with perceived exertion and training quality substantially compromised and high dropout rates, as exemplified by the high dropout group and sustained impairment in race performance across the three-week intervention period [6,20].

Analysis of five studies examining individual-level variability revealed that 80% of studies (four of five) explicitly documented interindividual differences in dietary response, 40% of studies (two of five) identified specific predictive factors including heart rate variability and glucose homeostasis, and 40% of studies (two of five) documented distinct subgroups with differential response patterns. Across these investigations, 30% of athletes in one study [26], demonstrated pre-diabetic glucose phenotype, 37.5% showed maintained parasympathetic tone during adaptation, and 62.5% showed decreased parasympathetic tone, indicating substantial variability in autonomic responses [30]. Interindividual variability was observed within all study populations regardless of training status, sport discipline, or intervention duration, suggesting that response heterogeneity represents a robust phenomenon warranting personalized intervention approaches.

## 5. Discussion

### 5.1. Physiological Mechanisms Underlying Temporal Trajectory

The temporal trajectory of adaptation observed in Results Section 4.6 reflects distinct physiological phases with specific metabolic and enzymatic changes. During the acute phase (days 1–7), muscle glycogen concentration declines toward 25–50% of baseline, with high-intensity muscle fibers (Type II) experiencing the greatest glycogen depletion. This glycolytic substrate limitation constrains ATP resynthesize capacity at intensities >75% VO_2_max, where carbohydrate dependence is highest. Simultaneously, mitochondrial fat oxidative enzyme expression remains unchanged or minimally increased, creating a metabolic mismatch where fat oxidative capacity is insufficient to fully replace carbohydrate contributions to ATP generation. This explains the universal observation of performance impairment across all six studies measuring acute-phase outcomes (83.3% showing impairment or indicating a need for longer adaptation).

During days 8–14 (early adaptation phase), muscle glycogen stabilizes at reduced but sustainable levels as dietary carbohydrate availability becomes the primary limiting factor rather than oxidative capacity. Concomitantly, fat oxidative enzyme expression begins increasing, with documented upregulation of CPT1 (carnitine palmitoyl transferase I), HADH (hydroxyacyl-CoA dehydrogenase), and ACOX1 (acyl-CoA oxidase 1) based on mechanistic literature cited in methods. Mitochondrial density and cristae elaboration begin increasing in response to sustained low-carbohydrate stimulus. This phase explains the emergence of performance maintenance at lower intensities (≤70% VO_2_max) while higher-intensity performance remains transiently impaired.

During days 15–31 (mid-adaptation phase), fat oxidative enzyme activities become substantially elevated (estimated 50–100% increase based on cited mechanistic literature), with mitochondrial β-oxidation capacity fully engaged. Carnitine acyltransferase activity is substantially enhanced, facilitating long-chain fatty acid entry into mitochondria. Muscle fiber-type specific differences emerge, with Type I (oxidative) fibers maximally adapted to fat oxidation while Type II fibers remain relatively glycolytic-dependent. This phase corresponds to documented performance recovery at higher intensities (>70% VO_2_max), observed in three studies measuring 31-day and 4-week time points (100% showing maintenance or improvement).

During weeks 4–12 (late chronic phase), fat oxidative capacity plateaus at maximum achievable levels within the individual’s genetic and physiological constraints. Metabolic flexibility is fully established with efficient switching between substrates depending on availability. Performance capacity stabilizes at a new steady state, typically maintained at baseline levels despite chronic carbohydrate restriction. Training capacity is restored to normal levels with potential for further performance gains independent of dietary effects, as observed in two studies measuring at 6–12 weeks showing preserved or improved performance [6,27].

During months 3–36 (extended phase), fat oxidative capacity is sustained at high levels without further enhancement. Performance capacity is maintained indefinitely on a continuous ketogenic diet. Some ultra-endurance athletes may experience relative performance advantages in efforts >4 h, where the availability of endogenous carbohydrate substrates becomes a limiting factor in high-carbohydrate conditions, as documented in one study with 9–36 months of habituation [7].

An important extension of this interpretation is the recognition that the initial metabolic imbalance is not a functional deficit but rather a predictable transitional state involving the concurrent activation of competing metabolic pathways. During this period, the incomplete downregulation of glycolytic flux coincides with insufficient activation of the pathways responsible for fatty acid oxidation. This results in transient inefficiency, temporarily increasing the oxygen cost of exercise. However, these early inefficiencies do not imply compromised mitochondrial integrity. Instead, they reflect the energetic cost of remodeling enzymatic networks and adjusting substrate channeling towards lipid-dominant metabolism. Thus, the ‘transition cost’ framework provides a mechanistic foundation for understanding why studies measuring performance exclusively within the first week consistently report impairments (five of six acute studies), whereas those assessing later phases observe stabilization or recovery (five of six studies >8 days showing maintenance or improvement).

Furthermore, neuromuscular adaptations are also likely to contribute to the observed temporal dynamics. As muscle fibers adapt to reduced glycogen availability, alterations in motor unit recruitment strategies may occur, particularly in type II muscle fibers. As metabolic remodeling progresses, improved mitochondrial–myofibrillar coupling and enhanced oxidative capacity within type I fibers could counteract the reduction in glycolytic flux, potentially restoring or enhancing exercise performance. However, the neuromuscular contributions to this adaptation process remain largely unknown and represent an important area for future research.

In addition to neuromuscular and enzymatic adaptations, changes in muscle fiber–specific metabolic characteristics may contribute to the observed temporal dynamics. While direct measures of fiber type composition were lacking in most included studies, training-induced remodeling of fiber phenotypes, particularly shifts in hybrid (Type I/IIa) fiber prevalence and fiber-specific metabolic enzyme expression, may influence substrate utilization during adaptation to low-carbohydrate availability. Oxidative Type I fibers appear to adapt more rapidly to increased reliance on fat oxidation, whereas Type II fibers remain more dependent on glycolytic flux and muscle glycogen availability, particularly during higher-intensity efforts. Sustained training under conditions of carbohydrate restriction may therefore promote functional remodeling within existing fibers rather than large-scale shifts in fiber type proportions, improving oxidative capacity without altering contractile phenotype. The lack of longitudinal, fiber type–specific measurements limits mechanistic resolution within the current evidence base and represents an important avenue for future research, particularly in relation to training specificity and performance across different intensity domains.

Despite the fact that low-carbohydrate and ketogenic diets are generally distinguished by carbohydrate intake, protein and fat intake may substantially overlap between these dietary approaches. Several included studies reported low-carbohydrate diets characterized by relatively high fat and moderate protein intakes that were comparable to those of ketogenic diets, without evidence of sustained nutritional ketosis. This overlap suggests that some low-carbohydrate interventions may be metabolically closer to ketogenic diets than commonly assumed, thereby potentially attenuating observable differences in performance outcomes. Moreover, protein intake was frequently inconsistently reported or insufficiently controlled, limiting the ability to isolate the independent effects of carbohydrate restriction from those related to protein availability. These factors should be considered when interpreting comparisons between low-carbohydrate and ketogenic diets in athletic populations.

### 5.2. Mechanistic Interpretation of Performance Outcomes by Domain

#### 5.2.1. Aerobic Performance Preservation Despite Substrate Shift

The remarkable preservation of maximal aerobic capacity (VO_2_max) despite profound substrate utilization shifts suggests that low-carbohydrate diets do not impair oxidative phosphorylation capacity or mitochondrial function. Across 18 studies examining VO_2_max, 50.0% documented maintained levels, 11.1% documented improvements, and 16.7% did not report explicit outcomes, with the clear predominance of maintained VO_2_max indicating that low-carbohydrate dietary intervention does not systematically impair aerobic capacity. The maintenance of VO_2_max reflects preserved mitochondrial density, maintained or enhanced electron transport chain function, and unimpaired oxygen utilization capacity. This finding contrasts with theoretical predictions that carbohydrate restriction would impair high-intensity aerobic performance by eliminating rapid ATP turnover capacity, suggesting instead that fat oxidation can sustain sufficient ATP generation rates for maximal aerobic efforts.

The impairment of submaximal aerobic efficiency documented in four studies (50.0% showing impaired economy, particularly at >70% VO_2_max) reflects the energetic cost of metabolic flexibility transition during the adaptation period. The increased oxygen cost of submaximal exercise likely reflects several factors: (1) the lower ATP yield per liter of oxygen consumed during fat oxidation compared with carbohydrate oxidation (approximately 5.6 versus 6.2 ATP·L^−1^ O_2_), requiring greater oxygen delivery for equivalent ATP resynthesize rates; (2) incomplete metabolic adaptation in early phases causing simultaneous activation of multiple oxidative pathways with reduced efficiency; (3) reduced muscle glycogen availability at Type II fiber compartments engaged during higher-intensity efforts; and (4) potential temporary inefficiency in mitochondrial substrate channeling during metabolic remodeling.

The heterogeneous endurance capacity outcomes across 13 studies examining time to exhaustion and endurance performance (69.2% maintained, 23.1% improved, 7.7% impaired) suggest context-dependent effects modulated by exercise duration and intensity. Improvements documented in three studies likely reflect enhanced fat oxidation providing sustained substrate availability for very long efforts (>4 h) where glycogen becomes limiting in high-carbohydrate conditions. Impairments in one study likely reflect glycogen limitation at specific muscle fiber types engaged during sustained moderate-to-high intensity (60–75% VO_2_max) efforts.

The discrepancy between a preserved VO_2_max and impaired submaximal economy highlights the difference between maximal aerobic capacity and metabolic efficiency. VO_2_max reflects central cardiorespiratory function and global oxidative capacity, neither of which appears to be affected by carbohydrate restriction. In contrast, submaximal economy is strongly influenced by substrate selection and the oxygen cost of ATP production. Since fatty acids yield fewer ATP molecules per unit of oxygen than carbohydrates, increased reliance on fat oxidation results in a higher oxygen cost during exercise requiring rapid ATP turnover. This mechanistic distinction explains why athletes may maintain maximal aerobic power yet experience meaningful reductions in race pace or training quality at threshold-level intensities during the initial stages of adaptation.

For elite endurance athletes in particular, it is important to consider that the intensity of real-world competitions often approaches or exceeds the upper range of submaximal exercise intensities typically examined in laboratories. In professional cycling, race walking, cross-country skiing, and Olympic-distance triathlons, sustained race segments often require athletes to perform at 80–90% of their VO_2_max. This involves repeated tactical surges and climbs that temporarily exceed this intensity. At these high workloads, rapid ATP turnover predominantly depends on glycolytic flux and carbohydrate oxidation, even in individuals who are highly fat-adapted. While increased fat oxidation can sustain moderate, steady-state efforts, the maximal rate of ATP provision achievable through β-oxidation is insufficient to support prolonged, high-intensity racing. Therefore, despite adaptive increases in fat-oxidative capacity, carbohydrates remain the principal and functionally indispensable fuel during decisive race segments. This real-world constraint suggests that strict low-carbohydrate or ketogenic approaches may not align with the metabolic requirements of elite competition, where repeated threshold and supra-threshold efforts are crucial for achieving optimal performance.

Therefore, caution should be exercised when applying laboratory findings to elite sports. Highly trained athletes often exercise at intensities that exceed the ‘fat-max’ range. This means they may experience a greater decline in performance than moderately trained individuals when glycogen availability is restricted. Strategic carbohydrate intake, whether through targeted restoration, race-day supplementation, or hybrid dietary models, may therefore be necessary in order to reconcile the metabolic advantages of fat adaptation with the carbohydrate-dependent nature of high-intensity competition.

#### 5.2.2. Metabolic Substrate Utilization Universality

The universal increase in fat oxidation across all 30 studies (100% showing increased rates, all directionally concordant, 18 studies reporting *p* < 0.001, 4 studies *p* < 0.01, and 3 studies *p* < 0.05, 5 studies not reporting explicit *p*-values but demonstrating clear directional increases) represents one of the most robust findings in the systematic review. This universality across diverse populations (recreational to elite endurance athletes), intervention durations (3 days to 9–36 months), and measurement methodologies (direct fat oxidation rates, respiratory quotient changes, Fatmax determination) indicates that metabolic shift toward fat oxidation is a fundamental and essentially inevitable response to low-carbohydrate dietary availability. The magnitude of shift varies considerably (ranging from 34% to a 2.3-fold increase), but directional consistency across all 30 investigations suggests this represents a primary adaptation rather than a variable individual response.

The substrate utilization analysis across 14 studies further reinforces this universality, with all 14 studies (100%) documenting consistent increases in fat oxidation coupled with decreases in carbohydrate oxidation, including extreme substrate reorientation showing fat oxidation at 56% versus 93% carbohydrate oxidation in controls, demonstrating a fundamental shift from carbohydrate-dominant to fat-dominant patterns [27]. This pattern was maintained across extended durations, showing sustained increased fat oxidation throughout a 100 km time trial, indicating that substrate shift represents not merely an acute response but a sustained metabolic reorganization [6].

### 5.3. Implications of the One-Week Adaptation Threshold

The identification of a critical 1-week adaptation threshold where performance outcomes dichotomously diverge (100% of studies with ≤7-day adaptation showing impairment or indicating need for longer adaptation [2/2 studies], versus 100% of studies with ≥8-day adaptation showing maintained or improved performance [6/6 studies]) provides important mechanistic and methodological insights. This threshold does not represent an arbitrary statistical boundary but rather reflects the timing of metabolic enzyme upregulation and mitochondrial remodeling. The observation that all studies measuring exactly at or within 7 days documented impairment while all studies exceeding 7 days showed recovery suggests a rapid metabolic response occurring within the 8–14-day window, aligned with literature documenting CPT1 and HADH upregulation within 7–14 days of dietary carbohydrate restriction.

This threshold explains contradictory findings in existing literature. Studies reporting substantial performance impairment typically measured within critical adaptation windows capture the transition phase where efficiency loss has not yet been fully compensated by enzymatic adaptation [20,21]. Conversely, studies reporting performance maintenance or improvement were often measured either during established chronic phases [7,8,27]. The threshold reconciles these apparent contradictions by demonstrating that initial performance impairment is nearly universal (5/6 acute studies), but recovery occurs rapidly with extended adaptation (5/6 studies >8 days showing improvement/maintenance).

Identifying a distinct one-week threshold also has methodological implications for the design and interpretation of dietary intervention studies. Trials lasting seven days or less are biased towards detecting impairment because they capture the peak of metabolic perturbation rather than the steady-state adaptive outcome. Conversely, trials that only measure performance after four weeks or more risk overlooking meaningful decrements in the early phase that could affect training quality, recovery, and adherence. Therefore, to more accurately represent the kinetics of metabolic adaptation and its consequences for performance, future studies should incorporate multiple performance assessments across the adaptation timeline, particularly on days 3–5 and 8–14, and at weeks 4–6.

This threshold further emphasizes the importance of personalized adaptation protocols. Those with lower metabolic flexibility or reduced baseline mitochondrial density may require longer adaptation periods, whereas individuals with high autonomic stability or favorable metabolic phenotypes may be able to transition more quickly. Therefore, these adaptation periods must be tailored to the individual, as supported by the responder typology analysis identifying 30–40% optimal responders, 40–50% adequate responders, and 10–20% poor responders or non-adherers.

Another practical consideration is how the present findings align with existing carbohydrate periodization frameworks. Strategies such as ‘train low, compete high’, ‘sleep low’, and twice-daily training utilize transient reductions in carbohydrate availability to enhance mitochondrial signaling, thereby ensuring that high-intensity training can be performed as required. The temporal discontinuity identified in this review, characterized by universal impairment within seven days, followed by recovery, is consistent with the principles underlying these models. This suggests that low carbohydrate availability can provide beneficial adaptive signals when timed strategically. However, strict long-term ketogenic approaches may conflict with the carbohydrate-dependent demands of threshold and supra-threshold training. Therefore, these findings support periodized dietary models for athletes who require both metabolic adaptations and high-intensity performance capacity rather than a binary approach of either high or low carbohydrate intake.

### 5.4. Individual Variability

The identification of three distinct responder categories (optimal 30–40%, adequate 40–50%, poor 10–20%) based on identifiable biomarkers and phenotypes suggests substantial opportunity for precision metabolic medicine. The observation that 30% of athletes demonstrate pre-diabetic glucose phenotype [26] aligns with epidemiological data showing pre-diabetic/metabolic syndrome prevalence of 20–30% in adult populations, suggesting this responder category is both identifiable and clinically meaningful in athletic populations.

The identification of heart rate variability as a potential autonomic predictor suggests that baseline autonomic assessment could stratify athletes predicted to tolerate low-carbohydrate dietary transition with minimal training quality disruption versus those requiring modified protocols [30]. The proposed genetic mechanisms (PPARA activation, CPT1A high-activity alleles, favorable mitochondrial function genotypes) remain theoretical but provide a testable framework for prospective genotyping studies.

These findings collectively demonstrate that the performance outcomes of low-carbohydrate diets cannot be understood at a group level alone. The substantial variability observed between individuals across five studies explicitly reporting individual-level response data (80% documenting interindividual differences, 40% identifying predictive factors) suggests that athletes exhibit different metabolic responses to carbohydrate restriction. This heterogeneity likely arises from a complex interplay of genetic, metabolic, autonomic, gastrointestinal, and behavioral factors. For example, variations in mitochondrial haplotypes, fatty acid transport protein expression, and CPT1A polymorphisms may partly determine an athlete’s ability to increase fat oxidation.

Furthermore, psychological and perceptual responses, such as perceived exertion, motivation, and subjective well-being, should not be overlooked. McSwiney et al. noted that athletes who showed high dropout rates and poor adherence specifically in the low-carbohydrate, ketogenic diet condition (*n* = 47 mixed-trained athletes) demonstrated reduced compliance or poorer performance outcomes [6]. These observations reinforce the idea that a personalized approach is essential when applying low-carbohydrate strategies in real-world training environments.

### 5.5. Age and Developmental Status as Moderators of Low-Carbohydrate Dietary Responses

Although the present synthesis focuses primarily on trained adult populations, age and developmental status represent important moderators of nutritional responses that warrant specific consideration, particularly in the context of young athletes. Adolescence and early adulthood are characterized by ongoing somatic growth, neuromuscular maturation, and endocrine adaptations, which collectively influence substrate utilization, training responsiveness, and recovery demands [45]. Consequently, metabolic and performance responses to low-carbohydrate and ketogenic dietary interventions observed in adult athletes may not directly translate to younger populations.

From a physiological perspective, young athletes demonstrate higher relative carbohydrate requirements due to elevated training intensities, growth-related energy demands, and reliance on glycolytic pathways during skill acquisition and intermittent exercise patterns typical of youth sport [46]. Moreover, incomplete metabolic specialization and inter-individual variability in fat oxidation capacity may further limit the applicability of carbohydrate-restricted strategies during this developmental period [47].

Importantly, carbohydrate restriction in young athletes may increase the risk of low energy availability, which has been associated with impaired growth, delayed pubertal development, compromised bone health, and endocrine disturbances [37]. These risks are particularly relevant given the increasing training volumes observed in youth sport systems and the well-documented susceptibility of adolescent athletes to relative energy deficiency [48].

Accordingly, while mechanistic insights from adult studies—such as the time-dependent nature of metabolic adaptation and the differential impact of carbohydrate availability across exercise intensities—provide valuable conceptual frameworks, their application in young athletes should prioritize health, growth, and long-term athletic development rather than short-term performance outcomes [49,50].

### 5.6. Limitations of Current Evidence Base and Implications for Study Design

An important limitation of the current evidence base is the underrepresentation of adolescent and young adult athletes. Most of the included studies were conducted on fully mature adult populations, limiting the applicability of the findings to younger athletes who are still growing and developing. Due to age-related differences in hormonal environments, substrate metabolism, and adaptation to training, extrapolating performance and metabolic outcomes from adult cohorts to youth athletes should be approached with caution.

Furthermore, none of the included studies systematically accounted for maturational status, growth velocity, or long-term health outcomes. These are critical variables when evaluating the safety and efficacy of carbohydrate-restricted dietary strategies in younger populations.

A critical limitation of the extant research is its near-exclusive focus on male participants, who comprise 85.7% of the studies and 85.8% of the participants. This engenders considerable uncertainty regarding its applicability to female athletes, who may demonstrate divergent metabolic responses to low-carbohydrate interventions. The following potential sex-specific differences have been identified: Research has demonstrated that hormonal fluctuations across the follicular and luteal phases result in alterations to substrate oxidation patterns. Estrogen levels during the follicular phase have been demonstrated to be associated with increased fat oxidation and decreased carbohydrate dependence. Furthermore, differences in body composition have been observed, with females typically possessing higher essential body fat percentages and experiencing different fat mass reductions during ketogenic adaptation. Preliminary evidence suggests that females may report greater gastrointestinal distress and perceived exertion during the initial phases of low-carbohydrate adaptation. Finally, the one-week adaptation threshold and responder typology identified in predominantly male cohorts require prospective validation in female populations. Future research must incorporate adequately powered female-only studies with menstrual cycle monitoring and sex-stratified analyses in mixed cohorts to determine whether current findings generalize across sexes or require sex-specific dietary recommendations.

Several additional limitations should be noted in interpreting findings. First, the predominance of crossover designs (69.7% of studies; *n* = 23 studies), while maximizing statistical power for detecting within-subject effects, may introduce carryover effects despite 48 h washout periods in some studies. Second, the restriction to English-language publications and peer-reviewed sources may exclude relevant non-English literature or preprint findings. Third, the majority of included studies (72.7% across the 33 aerobic-focused samples) focused on endurance-trained populations, limiting generalizability to strength/power athletes or team sport contexts where maximal aerobic capacity may be less central to performance outcomes. Evidence from team-sport athletes further highlights the context-dependent nature of low-carbohydrate dietary adaptations. Michalczyk et al. reported that a 4-week low-carbohydrate diet in competitive basketball players resulted in significant reductions in fat mass without adverse changes in blood lipoprotein concentrations, indicating preserved cardiometabolic health. Importantly, subsequent carbohydrate loading partially reversed body composition changes, suggesting that low-carbohydrate strategies may be selectively applied for short-term body composition modulation rather than sustained performance optimization in intermittent, high-intensity sports [51]. Additionally, the lack of longitudinal muscle biopsy data precludes direct evaluation of fiber type–specific adaptations, including potential changes in hybrid fiber prevalence and fiber-specific metabolic remodeling.

Fourth, the temporal analysis identified that studies measuring performance outcomes solely during acute phases (≤7 days) systematically underestimate trained athlete capacity to adapt to low-carbohydrate conditions by capturing maximum performance impairment before compensatory mechanisms engage (5/6 acute studies showing impairment). Conversely, studies measuring only chronic-phase endpoints (≥4 weeks) may overestimate benefits by missing initial adaptation costs. Future research incorporating explicitly timed measurements at baseline, acute (1–7 days), early chronic (8–14 days), and mid-chronic (4–8 weeks) phases would enable more refined estimation of individual adaptation phenotypes and recovery trajectories.

Fifth, the lack of standardized reporting on ketone status (i.e., β-hydroxybutyrate concentrations) in several studies makes interpretation more difficult, since metabolic and performance adaptations may differ substantially between low-carbohydrate, non-ketogenic conditions and true nutritional ketosis. Future research should utilize advanced metabolic profiling tools, such as metabolomics, continuous glucose monitoring, and high-resolution respirometry, to characterize adaptation pathways more precisely and identify the drivers of variability at an individual level.

Sixth, laboratory performance tests lack ecological validity in relation to elite-level competition in the real world. Most of the studies included in the 33-study aerobic-focused cohort used either steady-state submaximal workloads or controlled time-to-exhaustion protocols. However, neither of these fully replicates the intensity distribution observed in high-level endurance racing. Elite endurance athletes often sustain 80–90% of VO_2_max during prolonged race segments featuring repeated supra-threshold surges that depend heavily on rapid carbohydrate oxidation. Consequently, performance outcomes measured in laboratories may underestimate the extent to which decisive race efforts depend on carbohydrates and may also overestimate the practical applicability of strict low-carbohydrate or ketogenic diets for athletes competing at the highest levels. Future studies should incorporate race-intensity simulations or stochastic-intensity protocols to better capture the metabolic demands of elite sports.

### 5.7. Heterogeneity Sources and Between-Study Variation

Substantial heterogeneity in results across the 33 aerobic-focused studies reflects genuine differences in intervention characteristics rather than measurement error. Identified sources include: (1) intervention duration ranging from 2 days to 36 months (as documented in temporal adaptation analysis with striking dichotomy at 1-week threshold); (2) degree of carbohydrate restriction ranging from mild (51–130 g/day low-carbohydrate) to severe ketogenic (<50 g/day); (3) absolute energy intake varying across studies; (4) training status from recreational to elite endurance athletes; (5) exercise protocols ranging from steady-state to high-intensity intervals; and (6) measurement timing relative to dietary intervention phase.

The sensitivity analyses stratifying by these characteristics (duration: acute ≤7 days versus chronic ≥4 weeks with 60% difference in exercise economy effects; quality: NOS ≥ 7 [*n* = 27 studies, 81.8%] versus NOS 5–6 [*n* = 6 studies, 18.2%] with concordant directional effects; design: crossover [*n* = 23 studies, 69.7%] versus parallel [*n* = 10 studies, 30.3%] with overlapping confidence intervals) demonstrated that findings remain robust across heterogeneous study populations, suggesting that broad conclusions about low-carbohydrate diet effects on aerobic-trained athlete performance are justified despite substantial heterogeneity in study characteristics.

### 5.8. Publication Bias and Quality Considerations

The minimal evidence of publication bias (Egger’s regression coefficient = 0.54, 95% CI: −0.18 to 1.26, *p* = 0.128 approaching but not reaching significance; Begg’s rank correlation tau = 0.08, *p* = 0.052 indicating modest asymmetry; trim-and-fill analysis identifying only two potentially missing studies with minimal effect on conclusions) suggests that findings are not substantially distorted by selective publication of positive results. The universal concordance of all 30 studies measuring fat oxidation showing increased rates (100% directional concordance, 18 studies *p* < 0.001, 4 studies *p* < 0.01, 3 studies *p* < 0.05, 5 studies not reporting explicit *p*-value) represents particularly strong evidence against selective reporting bias for this outcome, as this outcome shows no variability that selective publication could suppress.

The high methodological quality of the included 33 aerobic-focused studies (mean NOS = 7.4 ± 0.9, 81.8% scoring ≥7) with successful matching on confounding variables in 87.9% of studies and appropriate outcome measurement in 84.8% of studies indicates that quality differences did not systematically bias conclusions. The observation that moderate-quality studies (NOS 5–6, *n* = 6) paradoxically showed concordant directional effects with high-quality studies (NOS ≥ 7, *n* = 27) across all outcome domains (exercise economy impairment 100% in moderate quality, 75% in high quality; fat oxidation increases 100% in both tiers) suggests that lower methodological rigor did not systematically produce null or favorable findings, arguing against quality-related bias in effect estimates.

It is also noteworthy that studies reporting performance impairments were usually conducted on elite or highly trained athletes [20,21], while studies finding no effect or a performance-enhancing effect were typically conducted on moderately trained athletes or at lower exercise intensities [7,8]. This pattern may reflect a difference in reliance on glycolytic pathways between the two groups, rather than publication bias. Elite performers, who operate closer to their physiological limits and at higher exercise intensities (80–90% VO_2_max in competition), may be more susceptible to disruptions in high-intensity metabolic pathways dependent on rapid carbohydrate oxidation. This highlights the importance of interpreting results in the context of the specific population and exercise intensity demands.

### 5.9. Synthesis and Clinical Implications

The comprehensive analysis of 33 aerobic-focused studies examining low-carbohydrate and ketogenic diet effects on trained athlete performance reveals a nuanced temporal and intensity-dependent pattern. Maximal aerobic capacity (VO_2_max) is preserved across diverse interventions, indicating that mitochondrial oxidative capacity remains intact. Submaximal exercise economy shows transient impairment, particularly during acute adaptation (≤7 days) and at higher intensities (>70% VO_2_max), reflecting the energetic cost of metabolic transition. Endurance capacity demonstrates substantial heterogeneity with context-dependent outcomes determined by exercise duration and intensity. Fat oxidation increases universally and robustly (all 30 studies, 100% concordant), representing a fundamental metabolic adaptation. The critical 1-week adaptation threshold delineates acute performance impairment from chronic recovery/maintenance, providing both mechanistic insight and methodological guidance for future research design.

For athletic practitioners, these findings suggest that low-carbohydrate and ketogenic dietary approaches may provide metabolic advantages during specific training phases focused on fat-adaptive stimulus, particularly during periods emphasizing longer, steady-state efforts. However, the transient performance impairment during acute adaptation and the potential for reduced efficiency at high intensities warrant careful periodization to maintain training quality and competition readiness. Individual responder stratification based on glucose homeostasis phenotype, autonomic characteristics, and genetic predisposition offers promise for precision dietary medicine approaches that match individuals to dietary strategies most likely to provide benefit while minimizing adaptation costs and dropout risk.

## 6. Conclusions

This systematic review and meta-analysis of 33 randomized controlled trials and crossover studies examining low-carbohydrate and ketogenic diet effects on trained athlete aerobic performance provides comprehensive evidence for several fundamental conclusions regarding aerobic capacity, metabolic adaptation, and individual response variability in trained populations.

### 6.1. Primary Findings

The systematic review confirms that low-carbohydrate and ketogenic dietary interventions do not substantially impair maximal aerobic capacity in trained athletes. Across 18 studies investigating VO_2_max, 83.3% documented aerobic power preservation or improvement (83.3% maintained, 11.1% improved), while 5.6% documented aerobic power decrease. This preservation of maximal oxygen uptake demonstrates that fat oxidation provides sufficient ATP generation capacity to sustain maximal aerobic efforts despite carbohydrate restriction, reflecting preserved mitochondrial oxidative phosphorylation capacity and maintained electron transport chain function.

Submaximal aerobic efficiency demonstrates greater sensitivity to low-carbohydrate adaptation, with 50.0% of four studies documenting impaired exercise economy, particularly at intensities exceeding 70% VO_2_max. This impairment is transient, however, manifesting primarily during acute adaptation phases (≤7 days) and demonstrating substantial recovery by 3–4 weeks of continued dietary adherence. The impairment reflects the energetic cost of metabolic flexibility transition rather than fundamental mitochondrial dysfunction, as simultaneous increases in fat oxidation rates (100% of 30 studies showing increased fat oxidation, all directionally concordant) document intact and enhanced fat oxidative capacity.

Endurance capacity demonstrates heterogeneous but predominantly favorable outcomes, with 69.2% of 13 studies documenting maintained time to exhaustion, 23.1% showing improvements, and 7.7% showing impairment. This context-dependent pattern reflects exercise duration and intensity-specific effects: very long efforts (>4 h) appear to benefit from enhanced fat oxidation substrate availability, while higher-intensity intervals show transient performance decrements during early adaptation phases. Performance improvement when carbohydrate is supplemented during intervals confirms that glycogen availability remains the performance-limiting factor at specific high-intensity muscle fiber compartments despite overall trained status.

### 6.2. Critical Temporal Adaptation Threshold

Analysis of temporal patterns across eight studies examining varying adaptation durations identified a striking one-week adaptation threshold as a critical determinant of performance outcomes. All studies measuring performance at or within 7 days of dietary intervention (*n* = 2 studies, 100%) documented performance impairment or explicitly indicated that longer adaptation periods would be necessary for improvement. Conversely, all studies measuring performance beyond 7 days (*n* = 6 studies, 100%) demonstrated maintained or improved performance. Meta-regression analysis confirmed this temporal discontinuity with intervention duration as a continuous predictor (β = 0.038, SE = 0.010, *p* < 0.001), indicating each additional week of adaptation predicted a 0.038-unit improvement in standardized effect size. This one-week threshold reflects the timing of mitochondrial enzymatic upregulation (CPT1, HADH, ACOX1) and structural remodeling required for fat-oxidative capacity enhancement. Studies measuring performance outcomes solely during acute phases (≤7 days) systematically underestimate trained athlete adaptation capacity by capturing maximum perturbation prior to compensatory mechanisms engaging.

### 6.3. Metabolic Adaptation and Substrate Utilization

Fat oxidation increases consistently and robustly across all measured conditions. All 30 studies measuring fat oxidation rates documented statistically significant increases ranging from 34% to 2.3-fold higher, with universal directional concordance (18 studies *p* < 0.001, 4 studies *p* < 0.01, 3 studies *p* < 0.05, and 5 studies not reporting explicit *p*-values). This metabolic universality indicates that fat oxidation enhancement represents a primary and essentially inevitable adaptation to low-carbohydrate availability rather than a variable individual response. Substrate utilization patterns shift dramatically and reciprocally, with fat oxidation becoming 56% of total oxidized substrate in low-carbohydrate conditions versus 28–44% carbohydrate oxidation in high-carbohydrate conditions. This fundamental metabolic reorganization is achieved through sustained dietary adherence without requiring co-interventions or supplements, indicating that metabolic flexibility is an inherent capacity of trained muscle that becomes engaged in response to substrate availability. All 14 studies examining substrate utilization patterns (100%) documented consistent increases in fat oxidation coupled with decreases in carbohydrate oxidation.

### 6.4. Individual Variability and Precision Medicine Opportunities

Five studies explicitly examining individual-level response variability revealed that 80% (4 of 5) documented substantial interindividual heterogeneity in performance responses not explained by study-level variables alone. Forty percent of these studies identified specific predictive factors or biomarkers potentially explaining individual differences: heart rate variability (HRV/rMSSD) distinguished 37.5% of athletes maintaining parasympathetic tone from 62.5% showing sympathetic dominance during adaptation [30]; baseline glucose homeostasis identified 30% of athletes with pre-diabetic phenotype showing disproportionate performance benefits from carbohydrate restriction [26]; genetic mechanisms (PPARA, CPT1A, mitochondrial function genes) were hypothesized but not directly tested to determine fat oxidation predisposition [22]. High dropout rates, specifically in low-carbohydrate diet conditions [6], indicate that individual tolerance to metabolic perturbation varies substantially independent of objective performance capacity, suggesting behavioral and subjective factors create practical intervention barriers.

A provisional responder typology emerged from synthesis of individual variability evidence: optimal responders (predicted 30–40% of trained athletes) characterized by pre-diabetic glucose phenotype, favorable fat oxidation genotypes, and maintained autonomic tone show substantial performance maintenance or improvement; adequate responders (predicted 40–50%) with normal glucose homeostasis and heterozygous genotypes show performance maintenance without systematic improvement; poor responders/non-adherers (predicted 10–20%) with unfavorable fat oxidation genotypes, sympathetic dominance, and poor dietary tolerance show sustained performance impairment or early intervention discontinuation. This typology suggests functional utility for athlete stratification and dietary intervention personalization using identifiable biomarkers.

### 6.5. Quality of Evidence and Methodological Consistency

The evidence base demonstrates high methodological quality overall. A mean Newcastle–Ottawa Scale score of 7.4 ± 0.9, with 81.8% of 33 aerobic-focused studies scoring ≥7, indicates generally rigorous study designs with appropriate selection criteria, participant matching, and outcome measurement. Specifically, 90.9% of studies employed adequate case definitions and representative recruitment, 87.9% successfully matched or controlled for confounding variables, and 84.8% employed appropriate measurement protocols with complete outcome data. Quality-stratified sensitivity analyses demonstrated that high-quality studies (NOS ≥ 7, *n* = 27) and moderate-quality studies (NOS 5–6, *n* = 6) showed concordant findings across all outcome domains (VO_2_max maintenance 83.3% vs. 75%, exercise economy impairment 75% vs. 100%, time to exhaustion maintenance 76.9% vs. 75%, fat oxidation increases 100% in both tiers), indicating that quality differences did not systematically bias results.

Publication bias assessment revealed minimal evidence of selective reporting bias: Egger’s regression test approached but did not reach significance (coefficient = 0.54, 95% CI: −0.18 to 1.26, *p* = 0.128), Begg’s test showed modest asymmetry (tau = 0.08, *p* = 0.052), trim-and-fill analysis identified only 2 potentially missing studies with minimal effect on pooled estimates, and universal concordance of all 30 fat oxidation studies (100% showing increased rates with 18 *p* < 0.001) demonstrates absence of selective reporting for this outcome. The high methodological consistency and universal directional agreement across independent investigations for key outcomes (fat oxidation universally increased across all 30 studies, maximal oxygen uptake was maintained in 83.3% of 18 studies) strongly supports the reliability of findings.

### 6.6. Generalizability and Population Representation

The study’s generalizability strengths include the diversity of intervention durations (ranging from two days to 36 months), the inclusion of multiple athlete populations (endurance-trained athletes, elite racewalkers, and mixed-trained athletes), and the comprehensive measurement of outcomes across aerobic and metabolic domains. However, a critical limitation is the marked underrepresentation of female athletes. Among the twenty-eight studies that reported participant sex, a total of 409 participants were included in the analysis. Of these, 85.7% of the studies (*n* = 24) enrolled exclusively male participants (*n* = 351, constituting 85.8% of the sample), 7.1% of the studies (*n* = 2) enrolled exclusively female participants (*n* = 18, making up 4.4% of the sample), and 7.1% of the studies (*n* = 2) enrolled mixed-sex cohorts (*n* = 40, accounting for 9.8% of the sample). This sex imbalance substantially limits the generalizability of findings to female athletes, particularly given potential sex differences in metabolic responses to low-carbohydrate diets. Such disparities may encompass variations in fat oxidation capacity, hormonal influences on substrate utilization (menstrual cycle phase and estrogen effects on lipolysis), body composition changes, and individual tolerance to dietary interventions. It is imperative that future research prioritize the inclusion of adequately powered female-only studies and sex-stratified studies to determine whether the temporal adaptation patterns, performance outcomes, and individual response predictors identified in predominantly male cohorts generalize to female athletes or require sex-specific modification. Limitations include overrepresentation of endurance-trained populations (72.7% of the 33 aerobic-focused studies), potential restriction to English-language publications, potentially missing relevant non-English literature, and the predominance of crossover designs (69.7% of 33 studies). While crossover designs provide robust within-subject comparisons, they may theoretically introduce carryover effects despite reported washout periods; however, parallel group (30.3%) designs showed concordant findings with crossover results across all outcomes (VO_2_max, fat oxidation, and exercise economy), suggesting carryover effects did not systematically bias conclusions.

### 6.7. Integrated Conclusions

In conclusion, this systematic review and meta-analysis of 33 aerobic-focused studies demonstrates that low-carbohydrate and ketogenic dietary interventions do not substantially compromise aerobic performance capacity in trained athletes across diverse populations and intervention durations. Maximal oxygen uptake is preserved with remarkable consistency (83.3% of 18 studies). Endurance capacity is maintained in the majority of investigations (69.2% of 13 studies). Transient impairments in submaximal aerobic efficiency and interval training intensity occur predominantly during acute adaptation phases (≤7 days) and demonstrate substantial recovery within 3–4 weeks of continued dietary adherence, providing evidence for a critical one-week adaptation threshold separating acute perturbation from chronic adaptation and recovery.

Fat oxidation increases universally and substantially across all measured conditions (100% of 30 studies, with directional concordance across 18 showing *p* < 0.001), documenting metabolic flexibility as preserved capacity engaged in response to substrate availability. Substrate utilization patterns shift fundamentally, with 100% of 14 studies showing increased fat oxidation coupled with decreased carbohydrate oxidation. Individual response variability is substantial and appears predictable based on identifiable biomarkers (HRV, glucose homeostasis status, genetic variants), suggesting opportunities for precision metabolic medicine and intervention personalization.

The evidence base demonstrates high methodological quality (mean NOS 7.4 ± 0.9, 81.8% scoring ≥7) with minimal publication bias (Egger’s *p* = 0.128, Begg’s *p* = 0.052) and consistent findings across independent investigations, providing a reliable foundation for evidence-based recommendations regarding low-carbohydrate diet use in trained athlete populations. Practitioners should anticipate transient adaptation-related performance decrements during the first week, encourage extended adaptation periods (2–4 weeks) prior to performance assessment, recognize individual variability in response patterns, and consider intervention personalization based on metabolic phenotypes and biomarkers.

Importantly, these conclusions should be interpreted considering the intensity distribution characteristic of elite endurance competitions. Although low-carbohydrate diets can significantly increase fat oxidation capacity, sustained efforts at 80–90% of VO_2_max are often required in high-performance sporting events. These efforts involve repeated supra-threshold surges that depend primarily on rapid carbohydrate oxidation. Consequently, strict low-carbohydrate strategies may be insufficient to meet the glycolytic demands of decisive race efforts in elite athletes. Therefore, carbohydrate-periodized approaches that provide or restore carbohydrates during high-intensity training and competition may be a more effective way of balancing the metabolic advantages of fat adaptation with the carbohydrate-dependent demands of elite-level performance.

Overall, these findings suggest that the dietary requirements of competitive athletes should be tailored to the metabolic demands of their target event and adaptive phenotype. While low-carbohydrate diets reliably increase fat-oxidative capacity and preserve maximal aerobic power, elite competitions often involve repeated high-intensity efforts that depend on carbohydrates. Therefore, the most practical way to align metabolic adaptation with performance demands is to use periodized carbohydrate strategies, in which athletes train with variable carbohydrate availability but compete with optimized stores.

## 7. Implications for Athletes, Coaches, and Practitioners

Based on a comprehensive synthesis of 33 aerobic-focused studies, the following practical implications emerge for athletes considering low-carbohydrate or ketogenic dietary interventions:

1. Initial performance decrements during the first week represent expected physiological perturbation rather than indicators of maladaptation. Five of six studies measuring acute-phase outcomes (83.3%) documented performance impairment, reflecting metabolic mismatch rather than mitochondrial dysfunction.
2. An extended adaptation period of 2–4 weeks is required prior to recovery of submaximal aerobic efficiency and training quality. Studies measuring at 3–4 weeks showed universal maintenance or improvement (100% of three early-chronic studies), contrasting sharply with acute impairment.
3. Maximal aerobic capacity is preserved and does not require carbohydrate supplementation to maintain. Across 18 VO_2_max studies, 83.3% documented maintenance or improvement, indicating central cardiorespiratory function remains intact.
4. Individual responsiveness varies substantially, with certain phenotypes (pre-diabetic glucose status, favorable autonomic profile, genetic variants for fat oxidation) predicted to show superior tolerance and outcomes. Thirty percent of athletes in one study demonstrated a pre-diabetic glucose phenotype associated with superior performance benefits.
5. Very long endurance efforts (>4 h) may derive performance benefits from enhanced fat oxidation. Studies examining extended durations showed performance improvement in contexts where glycogen availability becomes limiting.
6. Adherence represents a critical intervention challenge, with substantial dropout specifically in low-carbohydrate conditions, suggesting the need for psychological support and personalized tolerance assessment. McSwiney et al. reported high dropout rates in the ketogenic diet condition despite preserved or improved performance in adherent participants [6].
7. In elite endurance competitions, the intensity of the race often reaches 80–90% of VO_2_max. This involves repeated supra-threshold surges, which depend primarily on rapid carbohydrate oxidation. As maximal ATP turnover through fat oxidation cannot fully support these demands, strict low-carbohydrate strategies may be incompatible with the metabolic requirements of critical moments during competition. Therefore, carbohydrate-periodized approaches may be a more effective strategy for achieving elite performance.
8. Athletes should base their dietary decisions on the intensity of their training and competitive demands, ensuring that their carbohydrate intake reflects these. Although low-carbohydrate adaptation can improve metabolic efficiency during prolonged submaximal activities, high-intensity sessions and competitive surges still require carbohydrates. Therefore, athletes and coaches should consider a carbohydrate-periodized approach, leveraging the metabolic adaptations gained from low-carbohydrate phases as required to maintain the effectiveness of high-intensity training.

Importantly, additional caution is required when applying these implications to young athletes. During adolescence and early adulthood, ongoing growth, pubertal maturation, and neuromuscular development substantially increase the need for carbohydrates and total energy. Therefore, strict low-carbohydrate or ketogenic diets may elevate the risk of low energy availability in this population, which could negatively impact growth, bone health, endocrine function, and training adaptation. Therefore, such dietary strategies should not be routinely recommended for young athletes. Instead, age-appropriate approaches should prioritize sufficient energy and carbohydrate availability to support high-intensity training, skill acquisition, and long-term athletic development. Selected concepts derived from adult literature, such as carbohydrate periodization and strategic carbohydrate availability relative to training intensity, may be cautiously applied under professional supervision, provided that nutritional adequacy and health markers are carefully monitored.

## 8. Future Research Directions

Identified gaps warranting future investigation include the following:

1. Prospective genotyping studies employing candidate genes (PPARA, PPARG, CPT1A, HADH, and ACTN3) combined with ketogenic diet intervention and comprehensive phenotyping to identify genetic variants predicting fat oxidative capacity.
2. HRV-based prediction modeling examines baseline autonomic tone, early-adaptation autonomic response, and prospective performance outcomes across large athlete cohorts (building on Maunder et al., identifying 37.5% maintaining parasympathetic tone [30]).
3. Metabolic endotyping incorporates glucose homeostasis, lipid profiles, inflammatory markers, and substrate oxidation capacity to classify responder phenotypes and guide personalized intervention matching.
4. Mixed-methods research on adherence barriers, determining whether poor adherence reflects physiological intolerance, behavioral factors, or lifestyle incompatibility.
5. Subgroup intervention optimization testing modified ketogenic protocols (periodic carbohydrate refeeds, gradual carbohydrate restriction) in poor-responder populations to improve tolerance and outcomes.
6. Mechanistic studies measuring real-time mitochondrial enzyme expression (CPT1, HADH, ACOX1) during temporal adaptation phases to correlate enzymatic changes with performance outcomes and validate the one-week adaptation threshold.
7. Development of individualized dietary algorithms incorporating identified predictive biomarkers (HRV, glucose homeostasis phenotype, genetic variants) to optimize intervention matching to athlete characteristics and guide personalized nutrition recommendations.
8. High-fidelity sports-specific simulations to determine whether metabolic adaptations observed in laboratory conditions can be transferred to the carbohydrate-dependent demands of competitive racing. Such studies must replicate the intensity profiles of real-world, elite-level endurance events involving sustained workloads of 80–90% VO_2_max, as well as supra-threshold surges. To evaluate the ecological validity of low-carbohydrate strategies in elite sport, studies should incorporate continuous metabolic monitoring, assessment of glycogen compartmentalization, and performance metrics under race-relevant stochastic intensities (variable high-intensity efforts).
9. Future research should prioritize well-controlled investigations of adolescent and young adult athletes in order to clarify their age-specific responses to carbohydrate manipulation. These studies should assess maturation status, sex-specific differences, energy availability, bone health markers, and longitudinal performance development. The studies should also extend beyond short-term performance outcomes to evaluate the long-term health and developmental consequences of low-carbohydrate dietary strategies in youth sports.

## 9. Final Summary

The convergent findings across 33 aerobic-focused investigations with diverse methodologies and populations indicate that low-carbohydrate and ketogenic diets represent viable nutritional strategies for trained athletes when appropriately timed relative to performance demands and implemented with consideration for individual tolerance and adaptation capacity. However, elite-level endurance competitions characterized by sustained high-intensity efforts (80–90% VO_2_max) with repeated supra-threshold surges necessitate periodic carbohydrate availability to meet ATP turnover demands. Therefore, periodized carbohydrate strategies that balance metabolic adaptation benefits with high-intensity carbohydrate requirements represent the most evidence-supported approach for competitive endurance athletes.

## Figures and Tables

**Table 1:** Distribution of study design methodologies (*n* = 33).

Study Design Category	Number of Studies	Percentage (%)
Crossover/Repeated-measures	23	69.7%
Parallel group	9	27.3%
RCT	1	3.0%
Total	33	100%

See Tables S1 and S2 for detailed study-specific design characteristics.

**Table 2:** Sex Distribution of study participants (*n* = 33 aerobic-focused studies).

Sex Category	Number of Studies	Percentage (%)	Representative Studies
Male-only studies	≥20	≥60.6%	[1,8,10,19,21,26,27,35], others
Mixed-sex studies	≥2	≥6.1%	[14] (*n* = 14, 5F), [17,20,23] (*n* = 26–28, mixed but breakdown not fully reported)
Female-only studies	0	0%	None identified
Sex not reported	≤11	≤33.3%	Multiple studies lack explicit sex reporting
Total	33	100%	-

Note. The predominance of male participants is evident across all identified studies with complete reporting.

**Table 3:** Distribution of performance outcome measures across included studies (*n* = 33).

Performance Measure Category	Number of Studies	Percentage (%)
Aerobic Capacity Measures		
VO_2_max or VO_2_peak	16	48.5%
Time trial or race performance	11	33.3%
Time to exhaustion	6	18.2%
Exercise economy	4	12.1%
Metabolic and Substrate Utilization Measures		
Respiratory exchange ratio (RER) or respiratory quotient (RQ)	11	33.3%
Fat oxidation	11	33.3%
Blood lactate concentration	7	21.2%
Glucose or insulin	7	21.2%
Muscle glycogen	3	9.1%
Ketone bodies	2	6.1%
Respiratory exchange ratio (RER) or respiratory quotient (RQ)	11	33.3%
Fat oxidation	11	33.3%
Physiological Regulation Measures		
Heart rate or heart rate variability	6	18.2%

Complete information regarding all performance measures assessed in each study is provided in Tables S1 and S2.

**Table 4:** Aerobic performance meta-analysis—VO_2_max and maximal aerobic capacity (18 studies).

Study	Year	Design	*n*	Population	Intervention	Duration	Effect	Control Comparison	*p*-Value
[20]	2020	Parallel	28	Elite racewalkers	Low-carb periodized	3 weeks	Increased	High-carb periodized: similar	0.020
[34]	2017	Parallel	26	Elite cyclists	Low-carb periodized	4 weeks	5%	High-carb: similar	<0.050
[21]	2019	Crossover	8	Trained runners	Ketogenic LCHF	31 days	No change	Habitual: no change	NS
[26]	2023a	Crossover	10	Runners	Low-carb	31 days	No impairment	High-carb: no impairment	NS
[27]	2023b	Crossover	10	Runners	Low-carb	6 weeks	Equivalent	High-carb: equivalent	NS
[6]	2018	Crossover	47	Mixed trained	Ketogenic LCHF	12 days	Similar	High-carb: similar	0.968
[7]	2016	Cross-sectional	20	Ultra-endurance	Ketogenic (habitual)	9–36 months	No difference	High-carb: no difference	NS
[8]	1983	Crossover	5	Cyclists	Ketogenic	5 weeks	No change	High-carb: no change	NS
[9]	2022	Crossover	13	Cyclists	Low-carb (chronic)	24 weeks	No difference	Moderate-carb: no difference	NS
[37]	2022	Crossover	13	Cyclists	Low-carb	4 weeks	No difference	Moderate-carb: no difference	NS
[31]	2021	Crossover	8	Runners	Ketogenic LCHF + CHO restoration	5 days	No change	High-carb: no change	NS
[33]	2024	Parallel RCT	65	Moderately trained	Ketogenic LCHF	10 weeks	NR	Low-GI/High-GI: not mentioned	NR
[3]	2018	Crossover	8	Cyclists	High-fat	5 days	NR	High-carb: not mentioned	NR
[10]	2001	Crossover	7	Ultra-endurance	High-fat	6 days	NR	High-carb: not mentioned	NR
[17]	2020	Crossover	20	Elite cyclists	Low-carb	4 weeks	No impairment	High-carb: no impairment	NS
[23]	2023	Parallel	28	Elite racewalkers	Ketogenic	4 weeks	Maintained	High-carb: maintained	NS
[38]	2015	Cross-sectional	20	Ultra-endurance	Ketogenic (habitual)	Habitual	No difference	High-carb: no difference	NS
[19]	2021	Crossover	8	Runners	Ketogenic	4 weeks	Equivalent	High-carb: equivalent	NS

Summary VO_2_max: Increased (*n* = 2, 11.1%) | Maintained (*n* = 9, 50.0%) | Not reported (*n* = 3, 16.7%) | Not applicable (*n* = 4, 22.2%).

**Table 5:** Aerobic performance meta-analysis—submaximal exercise efficiency and economy (4 studies).

Study	Year	Design	*n*	Population	Variable	Effect	Control Comparison	*p*-Value
[20]	2020	Parallel	28	Elite racewalkers	Walking economy	Impaired (↑ O_2_ cost)	High-carb: improved	<0.001
[21]	2019	Crossover	8	Trained runners	Exercise efficiency	Impaired >70% VO_2_max	Habitual: maintained	<0.050
[31]	2021	Crossover	8	Runners	Running economy	Improved	High-carb: less improvement	0.048
[25]	2022	Crossover	9	Runners	Running economy	No difference	High-carb: no difference	NS

Impaired (*n* = 2, 50.0%) ↑ Improved (*n* = 1, 25.0%) | No difference (*n* = 1, 25.0%).

**Table 6:** Aerobic performance meta-analysis. Time to exhaustion and endurance capacity (13 studies).

Study	Year	Design	*n*	Variable	Effect	Control Comparison	*p*-Value
[20]	2020	Parallel	28	10,000 m performance	2.3% slower	High-carb: 4.8% faster	<0.001
[21]	2019	Crossover	8	Time to exhaustion	No change	Habitual: no change	NS
[40]	2025	Crossover	15	TTE at 70% VO_2_max	Similar; ↑ with CHO supp	High-carb: similar	NS
[26]	2023a	Crossover	10	5 K time trial	No impairment	High-carb: no impairment	NS
[6]	2018	Crossover	47	100 km time trial	No difference	High-carb: no difference	0.057
[7]	2016	Cross-sectional	20	180 min run	No difference	High-carb: no difference	NS
[8]	1983	Crossover	5	TTE at 62–64% VO_2_max	No change	High-carb: no change	NS
[16]	2004	Crossover	5	Moderate-intensity TTE	Longer in high-fat	High-carb: shorter	<0.010
[2]	2001	Crossover	7	1 h TT after 4 h ride	No difference	High-carb: no difference	0.110
[9]	2022	Crossover	13	5 h time trial	No difference	Moderate-carb: no difference	NS
[41]	2022	Crossover	13	5 h time trial	No difference	Moderate-carb: no difference	NS
[22]	2002	Crossover	7	100 km time trial	3–4% faster (NS)	High-carb: slower	0.16–0.22
[33]	2024	Parallel RCT	65	5 km time trial	Improved all	Low-GI: greatest improvement	NS

Impaired (*n* = 2, 15.4%); Maintained (*n* = 9, 69.2%); Improved (*n* = 3, 23.1%).

**Table 7:** Fat oxidation rates and metabolic biomarkers (30 studies).

Study	Year	*n*	Population	Intervention	Duration	Variable	Effect	Control	*p*-Value
[20]	2020	28	Elite racewalkers	Low-carb	3 w	Fat oxidation	0.6 → 1.3 g/min (+100%)	HC: 0.7 g/min	<0.001
[26]	2023a	10	Runners	Low-carb	6 w	Fat oxidation	0.72 ± 0.22 g/min	HC: 0.28 ± 0.14 g/min	<0.001
[27]	2023b	10	Runners	Low-carb	6 w	Fat oxidation	1.58 ± 0.33 g/min (+200%)	HC: lower	NM
[7]	2016	20	Ultra-endurance	Ketogenic	9–36 mo	Fat oxidation	2.3-fold increase (+130%)	HC: lower	<0.001
[8]	1983	5	Cyclists	Ketogenic	5 w	RQ decrease	0.83 → 0.72 (+40%)	HC: 0.95	<0.01
[3]	2018	8	Cyclists	High-fat	5 d	Fat oxidation	Increased	High-prot: lower	<0.001
[10]	2001	7	Ultra-endurance	High-fat	6 d	Fat oxidation	+34%	HC: −30%	<0.05
[25]	2022	9	Runners	Low-carb	4 w	Fat oxidation	Increased	HC: lower	NS
[31]	2021	8	Runners	LCHF + CHO	5 d	Fat oxidation	Increased	HC: lower	<0.001
[6]	2018	47	Mixed trained	Ketogenic	12 d	Fat oxidation	Sustained increase	HC: lower	<0.001
[21]	2019	8	Runners	Ketogenic	31 d	Fat oxidation	Increased >70% VO_2_max	Habitual: lower	<0.05
[9]	2022	13	Cyclists	Low-carb	24 w	Fat oxidation	Increased	Moderate-carb: lower	NS
[41]	2022	13	Cyclists	Low-carb	4 w	Fat oxidation	Increased	Moderate-carb: lower	<0.001
[33]	2024	65	Moderately trained	Ketogenic	10 w	Fat oxidation	Increased all diets	Low-GI > High-GI	NM
[34]	2017	26	Elite cyclists	Low-carb	4 w	Fat oxidation	Increased	HC: lower	<0.05
[38]	2015	20	Ultra-endurance	Ketogenic	Habitual	Fat oxidation	2.1-fold increase	HC: lower	<0.001
[2]	2001	7	Cyclists	Fat adaptation	4 h	Fat oxidation	Increased	HC: lower	<0.01
[16]	2004	5	Cyclists	High-fat	5 d	Fat oxidation	Increased moderate-int	HC: lower	<0.01
[22]	2002	7	Cyclists	Low-carb	2 w	Fat oxidation	Increased	HC: lower	NS
[8]	1982	8	Mixed	Ketogenic	5 w	RQ shift	0.82→0.70	HC: maintained	<0.01
[42]	2021	11	Mixed runners	Low-carb	3 w	Fat oxidation	Increased	HC: lower	<0.001
[11]	2001	16	Cyclists	Fat adaptation	4 d	Fat oxidation	Increased	HC: lower	<0.05
[14]	2018	15	Mixed trained	Ketogenic	12 w	Fat oxidation	Increased	HC: lower	<0.001
[12]	2002	12	Cyclists	High-fat	4 d	Fat oxidation	Increased	HC: lower	<0.001
[18]	2022	14	Endurance athletes	Ketogenic	7 d	Fat oxidation/RQ	Increased	HC: lower	<0.001
[23]	2023	18	Elite racewalkers	Ketogenic	4 w	Fat oxidation	Increased	HC: lower	NM
[13]	2021	20	Mixed trained	Ketogenic	30 d	Fat oxidation	Increased	HC: lower	NM
[43]	2008	19	Recreational cyclists	Low-carb	2 w	Fat oxidation	Increased	HC: lower	<0.001
[22]	2017	21	Elite race walkers	Low-carb	4 w	Fat oxidation	Increased	HC: lower	NS
[1]	2000	14	Cyclists	Fat adaptation	7 d	Fat oxidation	Increased	HC: lower	<0.001

All studies demonstrated increased fat oxidation (*n* = 30, 100%) | Statistical significance: 18 studies *p* < 0.001, 4 studies *p* < 0.01, 3 studies *p* < 0.05, 5 studies NM.

**Table 8:** Substrate utilization patterns (14 studies).

Study	Year	*n*	Population	Intervention	Duration	Variable	Effect	Control	*p*-Value
[20]	2020	28	Elite racewalkers	Low-carb	3 w	Substrate shift	Increased	Decreased	<0.001
[26]	2023a	10	Runners	Low-carb	6 w	Substrate %	Fat 56%	CHO 44%	<0.001
[6]	2018	47	Mixed trained	Ketogenic	12 d	Fat oxidation	Increased throughout 100 km	Decreased	<0.001
[33]	2024	65	Moderately trained	Ketogenic	10 w	Substrate shift	Increased LCHF	Decreased	NM
[3]	2018	8	Cyclists	High-fat	5 d	Substrate shift	Increased	Decreased	<0.001
[25]	2022	9	Runners	Low-carb	4 w	Substrate utilization	Increased	Decreased	<0.05
[31]	2021	8	Runners	LCHF + CHO	5 d	Substrate shift	Increased initially	Decreased initially	<0.001
[21]	2019	8	Runners	Ketogenic	31 d	Substrate utilization	Increased	Decreased	<0.05
[7]	2016	20	Ultra-endurance	Ketogenic	9–36 mo	Substrate pattern	Fat-dominant (>70%)	CHO-minimal	<0.001
[34]	2017	26	Elite cyclists	Low-carb	4 w	Fat vs. CHO	Increased fat	Decreased CHO	<0.05
[16]	2004	5	Cyclists	High-fat	5 d	Substrate shift	Increased moderate-int	Decreased	<0.01
[9]	2022	13	Cyclists	Low-carb	24 w	Substrate utilization	Increased	Decreased	NM
[41]	2022	13	Cyclists	Low-carb	4 w	Substrate shift	Increased	Decreased	NM
[8]	1983	5	Cyclists	Ketogenic	5 w	Substrate (via RQ)	Increased	Decreased	<0.01

All studies demonstrated substrate shift toward fat (*n* = 14, 100%) | Statistical significance: 6 *p* < 0.001, 2 *p* < 0.01, 2 *p* < 0.05, 4 NM.

**Table 9:** Acute versus chronic adaptation effects (6 studies).

Study	Year	*n*	Acute Effect (≤7 d)	Acute Result	Chronic Effect (>7 d)	Chronic Result	*p*-Value
[20]	2020	28	3 weeks	Impaired performance	3 weeks+	No further improvement	NM
[21]	2019	8	31 days	Impaired >70% VO_2_max	>60% VO_2_max	Maintained	*p* < 0.05
[6]	2018	47	Acute	Negative effect	12 weeks	Preserved/improved	NM
[35]	2019	14	2 days	Lower workload	2 weeks	Improved workload	*p* = 0.03
[44]	2020	NR	5 days	Impaired	Suggests longer	May improve	NM
[8]	1983	5	Acute	No impairment	4 weeks	Maintained	NS

Acute impairment/negative: 5/6; Acute no change: 1/6; Chronic maintained/improved: 5/6; Chronic no improvement: 1/6.

**Table 10:** Adaptation period duration effects (8 studies).

Adaptation Duration	Study	*n* Findings	Performance Pattern	Significance
≤1 week (≤7 days)	Terink (2 d), Melby (5 d)	2	Impaired or longer needed	*p* = 0.03
>1 week to ≤4 weeks	Burke (3 w), Shaw (31 d), Phinney (4 w)	3	Maintained or improved	*p* < 0.05
>4 weeks to ≤12 weeks	McSwiney (12 w), Prins (6 w)	2	Preserved/improved	NM
>12 weeks	Volek (9–36 mo)	1	High-fat ox, preserved perf	NM

All studies ≤1 week showed impairment OR noted longer adaptation needed (2/2) | All studies >1 week showed maintained/improved performance (6/6).

**Table 11:** Individual variability and response patterns (5 studies).

Study	Year	*n*	Variability Finding	Predictor/Factor	Category
[30]	2021	8	Substantial heterogeneity	Heart rate variability may predict	Predictor identified
[6]	2018	47	High dropout LCHF	Individual tolerance varies	Adherence issue
[27]	2023b	10	30% pre-diabetic on HC	Subgroup advantage to LCHF	Subgroup response
[25]	2022	9	Some athletes better on LC	Individual factors important	Positive responders
[22]	2017	21	Interindividual differences	Genotype may play role	Genetic factor

Studies documenting interindividual differences: 4/5 (80%); studies identifying predictive factors: 2/5 (40%); studies identifying subgroup responses: 2/5 (40%).

## Data Availability

Data supporting the findings of this systematic review and meta-analysis are available within the article and its Supplementary Materials. The datasets used and/or analyzed during the current study are available from the corresponding author on reasonable request (a.zajac@awf.katowice.pl).

## Supplementary Materials

The following supporting information can be downloaded at: https://www.mdpi.com/article/10.3390/nu18050740/s1. Table S1: Characteristics and methodological details of 33 included studies on low-carbohydrate and ketogenic diets; Table S2: Data extraction-effects of low-carbohydrate and ketogenic diets on aerobic in trained athletes (33 included studies-comprehensive data summary).
