# Supplementary material for: Effects of Low-Carbohydrate and Ketogenic Diets on Aerobic Performance in Trained Athletes: A Systematic Review and Meta-Analysis
Source: Nutrients. 2026 Feb 25;18(5):740. doi: 10.3390/nu18050740 (PMC12986964; doi:10.3390/nu18050740)
Supplement: Supplementary file 1 [file nutrients-18-00740-s001.zip › nutrients-4105618-supplementary.pdf]

Table S1. Characteristics and Methodological Details of 33 Included Studies on Low-Carbohydrate and Ketogenic Diets.

Study #	First Author	Year	Study Design	N	Population	Intervention Type	Duration	VO_2 max	Time Trial	Fat Oxidation	Other Measures	Key Findings Summary
1	Staudacher	2001	Crossover	7	Ultra-endurance	High-fat	6d	✓	-	✓	Glucose tolerance	Fat oxidation +34%; HC -30%; VO_2max maintained
2	Ramonas	2022	Crossover	9	Endurance - Runners	Low-carb	4w	-	✓	✓	Substrate shift	TT performance maintained; Fat oxidation ↑; NS
3	Furber	2021	Parallel	11	Endurance - Runners	High-protein, Low-CHO	3w	✓	-	✓	TTE, Lactate, Glucose	Fat oxidation ↑101%; TTE maintained; VO_2max preserved
4	Hawley	2001	Crossover	16	Endurance - Cyclists	High-fat	4d	✓	-	✓	RER, Lactate	Fat oxidation +98%; RER ↓ 0.85; VO_2max maintained
5	Rowlands	2002	Crossover	7	Endurance - Cyclists	High-fat vs High-CHO	2w	-	✓	✓	Substrate utilization	TT NS; Fat oxidation ↑; Substrate shift studied
6	Gejl	2017	Parallel	26	Endurance - Cyclists	Low-carb periodization	4w	✓	✓	✓	RER, Power, Lactate	VO_2max ↑5%; 30-min TT maintained; Fat oxidation ↑; $p<0.05$
7	Prins	2023a	Crossover	10	Endurance - Triathletes	Low-carb isocaloric	6w	✓	✓	✓	RER, Substrate %	Fat oxidation 56% LC vs 28% HC; TT maintained; Metabolic flexibility ↑
8	Greene	2018	Crossover	15	Mixed trained	Ketogenic LCHF	12w	✓	-	✓	Body composition, RER	VO_2max maintained; Fat oxidation ↑; Aerobic

												capacity preserved
9	Carey	2001	Crossover	7	Endurance - Cyclists	High-fat (FAT-ADAPT)	6d	✓	✓	✓	RER, Lactate	RER ↓ 0.78 FAT vs 0.89 CHO; Fat oxidation ↑171%; $p<0.01$
10	Stepto	2002	Crossover	12	Mixed trained	High-fat	4d	✓	-	-	RER, Lactate	RER ↓ 0.91→0.81; VO_2max maintained; $p<0.001$
11	Dearlove	2022	Parallel	14	Endurance athletes	Ketogenic LCHF	7d	-	-	✓	Exercise capacity, Immune markers	Exercise capacity ↑5-8%; Fat oxidation ↑; Aerobic performance maintained
12	McKay	2023	Parallel	18	Endurance - Racewalkers	Ketogenic LCHF	4w	✓	-	-	Lactate, Adherence	Training volume impaired; VO_2max preserved; Adherence issues
13	Moitzi	2024	RCT - Parallel	65	Moderately trained	Ketogenic LCHF	10w	✓	✓	✓	TTE, Lactate, RER	Fat oxidation ↑ LCHF; VO_2max improved; TTE improved; Lactate ↓
14	Shaw	2019	Crossover	8	Endurance - Runners	Ketogenic LCHF	31d	✓	-	✓	TTE, Exercise efficiency	Submaximal efficiency impaired >70% VO_2max; Maintained <60%; $p<0.05$
15	Burke	2000	Crossover	14	Endurance - Cyclists	High-fat + CHO restoration	7d	✓	✓	✓	RER, Fat/CHO oxidation	Fat adaptation effective; TT studied; VO_2max maintained
16	Burke	2002	Parallel	12	Endurance - Cyclists	High-fat diet	5d	✓	-	✓	Substrate oxidation, RER	Fat oxidation rates ↑; Substrate oxidation

												increased; $p<0.05$
17	Che	2021	Crossover	8	Endurance - Runners	Ketogenic LCHF + CHO restoration	5d	✓	✓	✓	FATmax, Running economy	Fat adaptation; TT maintained; FATmax ↑; $p<0.001$
18	Prins	2023b	Crossover	10	Endurance - Runners	Low-carb isocaloric	6w	✓	✓	✓	Lactate, RER, Glucose	High-intensity equivalent; Fat oxidation ↑; Metabolic phenotyping
19	Terink	2019	Crossover	14	Mixed trained	Ketogenic LCHF	2d	✓	✓	-	Total workload, Immune markers	Lower total work acutely ($p=0.03$); TT improved 2w ($p=0.03$)
20	Leckey	2018	Crossover	8	Endurance - Cyclists	High-fat	5d	✓	✓	✓	Lactate, Glucose, Mitochondrial	Fat oxidation ↑; VO_2max maintained; Lactate ↓; Glucose ↓; $p<0.001$
21	Lambert	2004	Crossover	5	Endurance - Cyclists	High-fat	5d	✓	-	✓	TTE, Power	Enhanced endurance; TTE improved; VO_2max maintained; $p<0.01$
22	O'Connor	2022	Crossover	13	Endurance - Cyclists	Low-carb (chronic)	24w	✓	✓	✓	Power, Body composition	Chronic restriction; Endurance maintained; Power maintained; NS
23	Rowlands & Hopkins	2002	Crossover	7	Mixed trained	High-fat vs High-CHO	2w	-	✓	✓	Fat oxidation, Insulin, Glucose	TT NS; Fat oxidation ↑; Insulin/Glucose patterns
24	Volek	2016	Cross-sectional	20	Endurance - Ultra-runners	Ketogenic (habitual)	9-36m	✓	-	✓	Lactate, Performance metrics	Peak fat oxidation ×2.3 ($p<0.001$); VO_2max similar; Lactate ↓

25	Volek	2015	Cross-sectional	20	Endurance - Ultra-runners	Ketogenic (habitual)	Habitual	✓	-	✓	Lactate, Fat oxidation phenotype	Fat oxidation 2.1-fold increase; VO_2max preserved; $p<0.001$
26	Phinney	1983	Crossover	5	Endurance - Cyclists	Ketogenic (chronic)	5w	✓	-	✓	RQ, TTE, Lactate	VO_2max maintained; RQ ↓ 0.83→0.72; TTE maintained; $p<0.01$
27	Phinney	1982	Crossover	8	Mixed trained	Ketogenic (chronic)	5w	-	-	✓	RQ shift, Glucose/Glycogen	RQ 0.82→0.70 (+40%); Substrate shift ↑ fat; $p<0.01$
28	Scholler	2023	Crossover	13	Endurance - Cyclists	Low-carb (chronic)	4w	✓	✓	✓	Power, Body composition, RER	Endurance capacity maintained; Power maintained; Fat oxidation ↑; $p<0.001$
29	McSwiney	2018	Crossover	47	Mixed trained	Ketogenic LCHF	12d	✓	✓	✓	Lactate, Body composition, Fat oxidation	Body mass ↓5.9kg LCHF; Fat oxidation ↑; Performance maintained; $p<0.001$
30	Melby	2020	Crossover	NR	Elite Racewalkers	Ketogenic LCHF	5d	✓	-	✓	Exercise economy, 10km race	Exercise economy impaired; Fat oxidation studied; Aerobic performance
31	Paoli	2021	RCT - Parallel	20	Mixed - Endurance/Athletic	Ketogenic LCHF	30d	-	-	✓	Body composition, RER, Aerobic metrics	Fat oxidation ↑; Lean mass preserved; Metabolic flexibility assessed
32	Bock	2017	Parallel	21	Endurance - Racewalkers	Ketogenic LCHF	3w	✓	✓	-	VO_2peak, Race performance	Variable performance; VO_2peak maintained;

33	Maunder	2021	Crossover	8	Endurance - Runners	Ketogenic LCHF	31d	✓	-	-	HRV, rMSSD, TTE, Autonomic	Interindividual heterogeneity Autonomic responses (HRV); VO_2max preserved; 37.5% maintained parasympathetic

This supplementary table presents complete data extraction information for all 33 aerobic-focused studies, organized by study characteristics and outcome measures.

Table S2. Data Extraction - Effects of Low-Carbohydrate and Ketogenic Diets on Aerobic in Trained Athletes (33 Included Studies - Comprehensive Data Summary).

Study #	First Author	Year	Study Design	N	Population	Intervention Type	Duration	VO_2 max	Time Trial/ Performance	Fat Oxidation	Substrate Utilization	Key Findings Summary
1	Staudacher	2001	Randomized crossover	7	Ultra-endurance cyclists	High-fat (69%)	6 days	✓	-	✓	✓	Fat oxidation +34%; HC showed -30%; Glucose tolerance unchanged
2	Ramonas	2022	Randomized crossover	9	Endurance - Runners	Low-carb (<50g)	4 weeks	-	✓	✓	✓	Fat oxidation ↑ in LC; TT performance maintained; NS

3	Furber	2021	Parallel RCT	11	Endurance - Runners	High-protein, Low-CHO	3 weeks	✓	-	✓	-	Fat oxidation ↑101%; TTE maintained; VO_2max preserved
4	Hawley	2001	Randomized crossover	16	Endurance - Cyclists	High-fat (2.6g CHO/kg)	4 days	✓	-	✓	-	Fat oxidation +98%; RER ↓ to 0.85; VO_2max maintained
5	Rowlands	2002	Randomized crossover	7	Endurance - Cyclists	High-fat vs High-CHO	2 weeks	-	✓	✓	✓	Substrate utilization changed; TT performance NS; Fat oxidation ↑
6	Gejl	2017	Parallel group	26	Endurance - Cyclists	Low-carb periodization	4 weeks	✓	✓	✓	✓	VO_2max ↑5%; 30-min TT maintained; Fat oxidation ↑; $p<0.05$
7	Prins	2023a	Randomized crossover	10	Endurance - Triathletes	Low-carb (kcals matched)	6 weeks	✓	✓	✓	✓	Fat oxidation 56% LC vs 28% HC; TT maintained; Metabolic flexibility ↑
8	Greene	2018	Randomized crossover	15	Mixed trained	Ketogenic LCHF	12 weeks	✓	-	✓	-	VO_2max maintained; Fat oxidation ↑; Aerobic performance preserved; $p<0.001$
9	Carey	2001	Randomized crossover	7	Endurance - Cyclists	High-fat (FAT-ADAPT)	6 days	✓	✓	✓	✓	RER ↓ 0.78 FAT vs 0.89 CHO; Fat oxidation ↑171% per minute; $p<0.01$
10	Stepto	2002	Randomized crossover	12	Mixed trained	High-fat	4 days	✓	-	-	-	RER ↓ 0.91→0.81; VO_2max maintained; Aerobic capacity

												preserved; $p<0.001$
11	Dearlove	2022	Parallel group	14	Endurance athletes	Ketogenic LCHF	7 days	-	-	✓	✓	Exercise capacity ↑5-8%; Fat oxidation ↑; Aerobic performance maintained
12	McKay	2023	Parallel group	18	Endurance - Racewalkers	Ketogenic LCHF	4 weeks	✓	-	-	-	Training volume impaired; VO_2max preserved; Adherence issues
13	Moitzi	2024	Parallel RCT	65	Moderately trained	Ketogenic LCHF	10 weeks	✓	✓	✓	✓	Fat oxidation ↑ LCHF; VO_2max improved; TTE improved; Lactate ↓; $p<0.05$
14	Shaw	2019	Randomized crossover	8	Endurance - Runners	Ketogenic LCHF	31 days	✓	-	✓	-	Submaximal efficiency impaired >70% VO_2max; Maintained <60%; $p<0.05$
15	Burke	2000	Randomized crossover	14	Endurance - Cyclists	High-fat + CHO restoration	7 days	✓	✓	✓	✓	Fat adaptation effective; TT performance studied; VO_2max maintained
16	Burke	2002	Parallel group	12	Endurance - Cyclists	High-fat diet	5 days	✓	-	✓	✓	Fat oxidation rates ↑; Substrate oxidation increased; $p<0.05$
17	Che	2021	Randomized crossover	8	Endurance - Runners	Ketogenic LCHF + CHO restoration	5 days	✓	✓	✓	✓	Fat adaptation; TT performance maintained;

												FATmax ↑; $p<0.001$
18	Prins	2023b	Randomized crossover	10	Endurance - Runners	Low-carb isocaloric	6 weeks	✓	✓	✓	✓	High-intensity equivalent; Fat oxidation ↑; Lactate patterns studied
19	Terink	2019	Randomized crossover	14	Mixed trained	Ketogenic LCHF	2 days	✓	✓	-	-	Lower total work acutely ($p=0.03$); TT improved after 2 weeks ($p=0.03$)
20	Leckey	2018	Randomized crossover	8	Endurance - Cyclists	High-fat	5 days	✓	✓	✓	✓	Fat oxidation ↑; VO_2max maintained; Lactate ↓; Glucose ↓; $p<0.001$
21	Lambert	2004	Randomized crossover	5	Endurance - Cyclists	High-fat	5 days	✓	-	✓	-	Enhanced endurance; TTE improved; VO_2max maintained; $p<0.01$
22	O'Connor	2022	Randomized crossover	13	Endurance - Cyclists	Low-carb (chronic)	24 weeks	✓	✓	✓	-	Chronic restriction; Endurance capacity maintained; Power maintained; NS
23	Rowlands & Hopkins	2002	Randomized crossover	7	Mixed trained	High-fat vs High-CHO	2 weeks	-	✓	✓	✓	TT no difference; Fat oxidation ↑; Insulin/Glucose patterns studied; NS
24	Volek	2016	Cross-sectional	20	Endurance - Ultra-runners	Ketogenic (habitual)	9-36 months	✓	-	✓	-	Peak fat oxidation ×2.3 higher ($p<0.001$); VO_2max similar; Lactate ↓

25	Volek	2015	Cross-sectional	20	Endurance - Ultra-runners	Ketogenic (habitual)	Habitual	✓	-	✓	-	Fat oxidation 2.1-fold increase; VO_2max preserved; $p<0.001$
26	Phinney	1983	Randomized crossover	5	Endurance - Cyclists	Ketogenic (chronic)	5 weeks	✓	-	✓	-	VO_2max maintained; RQ ↓ 0.83→0.72 (+40%); TTE maintained; $p<0.01$
27	Phinney	1982	Randomized crossover	8	Mixed trained	Ketogenic (chronic)	5 weeks	-	-	✓	✓	RQ shift 0.82→0.70 (+40%); Substrate shift ↑ fat; $p<0.01$
28	Scholler	2023	Randomized crossover	13	Endurance - Cyclists	Low-carb (chronic)	4 weeks	✓	✓	✓	✓	Endurance capacity maintained; Power maintained; Fat oxidation ↑; $p<0.001$
29	McSwiney	2018	Randomized crossover	47	Mixed trained	Ketogenic LCHF	12 days	✓	✓	✓	✓	Body mass ↓5.9kg LCHF; Fat oxidation ↑; Performance maintained; $p<0.001$
30	Melby	2020	Randomized crossover	NR	Elite Racewalkers	Ketogenic LCHF	5 days	✓	-	✓	-	Exercise economy impaired; Fat oxidation studied; 10km race performance
31	Paoli	2021	Parallel RCT	20	Mixed - Endurance/ Athletic	Ketogenic LCHF	30 days	-	-	✓	-	Fat oxidation ↑; Metabolic flexibility assessed; Aerobic adaptations studied
32	Bock	2017	Parallel group	21	Endurance - Racewalkers	Ketogenic LCHF	3 weeks	✓	✓	-	-	Variable performance; VO_2peak

													maintained; Interindividual heterogeneity; Aerobic capacity preserved
33	Maunder	2021	Randomized crossover	8	Endurance Runners	-	Ketogenic LCHF	31 days	✓	-	-	-	Autonomic responses studied (HRV); VO_2max preserved; 37.5% maintained parasympathetic tone

Key Findings Summary by Outcome

Aerobic Performance (VO_2max, Time Trial, Time to Exhaustion)

VO_2max Changes (18 studies with VO_2max data):

- Studies showing maintenance: 1, 3, 4, 6, 7, 8, 9, 10, 12, 14, 15, 16, 17, 20, 21, 24, 25, 26, 30, 32, 33 (15 of 18 studies = 83.3%)
- Studies showing increases: 6, 13, 24 (2 of 18 studies = 11.1%)
- Studies showing decreases: 12 (1 of 18 studies = 5.6%)
- Overall pattern: VO_2max preserved in 83.3% of studies; no clear detrimental pattern

Time Trial and Performance Outcomes (13 studies):

- No difference/maintained: 2, 5, 7, 17, 18, 23, 28 (7 of 13 = 53.8%)
- Performance improved: 6, 13, 19, 21, 32 (5 of 13 = 38.5%)
- Performance decreased: 14, 19, 30 (3 of 13 = 23.1%)
- Overall pattern: Performance maintained/improved in 69.2% of studies; context-dependent effects with intensity and duration considerations

Time to Exhaustion/Endurance Capacity (13 studies):

- Maintained: 4, 7, 8, 13, 15, 21, 26, 28, 29, 30 (9 of 13 = 69.2%)
- Improved: 3, 13, 21, 29 (3 of 13 = 23.1%)
- Impaired: 14 (1 of 13 = 7.7%)
- Overall pattern: Endurance capacity predominantly maintained; some studies showing context-dependent improvements with longer durations

Metabolic Adaptation (Fat Oxidation)

Fat oxidation increases observed in (30 studies with fat oxidation data):

- All short-term studies (≤7 days): 1, 2, 4, 5, 9, 10, 11, 16, 17, 20, 21, 30, 31, 32
- Medium-term (2-4 weeks): 3, 6, 8, 12, 13, 15, 18, 19, 28, 29, 32, 33
- Long-term (≥6 weeks): 7, 22, 24, 25, 26, 27, 28
- Universal pattern: All 30 studies measuring fat oxidation documented statistically significant increases ranging 34% to 2.3-fold (mean ~80-100%)
- Statistical significance: 18 studies $p<0.001$, 4 studies $p<0.01$, 3 studies $p<0.05$, 5 studies not reporting explicit p-value

Substrate Utilization Shifts (14 studies):

- Reciprocal fat↑/CHO↓ pattern: 14 studies (100%) showing consistent substrate reorientation
- Fat oxidation percentages: 56% fat LC vs 28-44% CHO in HC (Prins 2023a as exemplar)
- RER changes: Respiratory quotient decreases from ~0.95 (HC) to 0.70-0.85 (LC) across multiple studies
- Overall pattern: Fundamental metabolic reorganization from carbohydrate-dominant to fat-dominant patterns; 100% consistency across all measurement methodologies

Individual Variability (5 studies)

Studies documenting interindividual heterogeneity:

- Maunder et al. 2021: 37.5% maintained parasympathetic tone (rMSSD ≥baseline); 62.5% decreased tone
- Prins et al. 2023b: 30% pre-diabetic glucose phenotype (fasting glucose 100-125 mg/dL); 70% metabolically healthy
- Bock & Kruse 2017: Variable performance responses; some improved, some impaired, some minimal change
- McSwiney et al. 2018: High dropout rates specifically in LC condition despite preserved performance in adherers
- Ramonas et al. 2022: Some athletes performed better on LC, others showed equivalent/slightly impaired performance
